# Supplementary material for: Risk factors for extended-spectrum beta-lactamase (ESBL)-producing E. coli carriage among children in a food animal-producing region of Ecuador: A repeated measures observational study
Source: PLoS Med. 2023 Oct 13;20(10):e1004299. doi: 10.1371/journal.pmed.1004299 (PMC10621961; doi:10.1371/journal.pmed.1004299)
Supplement: S1 Files — Checklist: Strengthening the Reporting of Observational Studies in Epidemiology (STROBE) checklist of items that should be included in reports of cohort studies. Sample Size and Power Calculations. Table A. Prevalence of third-generation cephalosporin-resistant, extended-spectrum beta-lactamase, multidrug-resistant, and extensively drug-resistant E. coli among children. Table B. Proportion of third-generation cephalosporin-resistant E. coli (3GCR-EC) isolates resistant to individual antibiotics in phenotypic susceptibility testing by data collection cycle. Table C. Antibiotic resistance of 3GCR-EC isolates from animal fecal samples (1 colony isolated per fecal sample) collected at the same households as child fecal samples, stratified by animal species. Table D. Prevalence of clinically important sequence types (ST) among sequenced 3GCR-EC isolates (N = 571) from child fecal samples. Table E. Proportion of 3GCR-EC isolates with beta-lactamase genes (among 15 most prevalent) detected in whole-genome sequences, stratified by phenotypic ESBL production. Table F. Prevalence of beta-lactamase resistance genes among sequenced 3GCR-EC isolates from children, stratified by parishes and intensity of commercial food animal production. Table G. Average number of total antibiotic resistance genes (ARGs) per third-generation cephalosporin-resistant E. coli isolate from children, stratified by parish. Table H. Prevalence of CTX-M-type genes among sequenced 3GCR-EC isolates from children, stratified by parish and intensity of commercial food animal production. Table I. Sensitivity analysis results for main analysis associations between combined food animal exposures and 3GCR-EC and ESBL-EC including only 1 isolate per child fecal sample. Table J. Associations between secondary risk factors and 3GCR-EC carriage among children. Table K. Associations between secondary risk factors and ESBL-EC carriage among children. Table L. Secular trends in caregiver-reported child illness and ant [file pmed.1004299.s001.docx]

# S1. Supporting Information File

####

**Checklist.** Strengthening the Reporting of Observational Studies in Epidemiology (STROBE) checklist of items that should be included in reports of cohort studies.

|  | **Item No** | **Recommendation** | **Section & Paragraph No** |
| --- | --- | --- | --- |
| **Title and abstract** | 1 | (*a*) Indicate the study’s design with a commonly used term in the title or the abstract | Abstract paragraph 2 |
|  |  | (*b*) Provide in the abstract an informative and balanced summary of what was done and what was found | Abstract paragraph 2 |
| **Introduction** | | | |
| Background/rationale | 2 | Explain the scientific background and rationale for the investigation being reported | Introduction paragraphs 1-3 |
| Objectives | 3 | State specific objectives, including any prespecified hypotheses | Introduction paragraph 4 |
| **Methods** | | | |
| Study design | 4 | Present key elements of study design early in the paper | Methods subsection Study Design |
| Setting | 5 | Describe the setting, locations, and relevant dates, including periods of recruitment, exposure, follow-up, and data collection | Methods subsections Study Site and Study Design; Supplemental materials Figure B |
| Participants | 6 | (*a*) Give the eligibility criteria, and the sources and methods of selection of participants. Describe methods of follow-up | Methods subsection Study Design |
|  |  | (*b*) For matched studies, give matching criteria and number of exposed and unexposed | NA |
| Variables | 7 | Clearly define all outcomes, exposures, predictors, potential confounders, and effect modifiers. Give diagnostic criteria, if applicable | Methods subsections Exposure Assessment and Outcome Assessment and Statistical Analysis |
| Data sources/ measurement | 8 | For each variable of interest, give sources of data and details of methods of assessment (measurement). Describe comparability of assessment methods if there is more than one group | Methods subsections Exposure Assessment and Outcome Assessment and Statistical Analysis |
| Bias | 9 | Describe any efforts to address potential sources of bias | Methods subsection Statistical Analysis |
| Study size | 10 | Explain how the study size was arrived at | Supplemental Materials Figure B |
| Quantitative variables | 11 | Explain how quantitative variables were handled in the analyses. If applicable, describe which groupings were chosen and why | Methods subsection Statistical Analysis |
| Statistical methods | 12 | (*a*) Describe all statistical methods, including those used to control for confounding | Methods subsection Statistical Analysis |
|  |  | (*b*) Describe any methods used to examine subgroups and interactions | Methods subsection Statistical Analysis |
|  |  | (*c*) Explain how missing data were addressed | Methods subsection Statistical Analysis |
|  |  | (*d*) If applicable, explain how loss to follow-up was addressed | Methods subsection Study Design |
|  |  | (*e*) Describe any sensitivity analyses | Methods subsection Statistical Analysis |
| **Results** | | |  |
| Participants | 13* | (a) Report numbers of individuals at each stage of study—eg numbers potentially eligible, examined for eligibility, confirmed eligible, included in the study, completing follow-up, and analysed | Supplemental Materials Figure B |
|  |  | (b) Give reasons for non-participation at each stage | Supplemental Materials Figure B |
|  |  | (c) Consider use of a flow diagram | Supplemental Materials Figure B |
| Descriptive data | 14* | (a) Give characteristics of study participants (eg demographic, clinical, social) and information on exposures and potential confounders | Results subsections Household & Child Characteristics, Food Animal Production & Domestic Animals, Tables 1 and 2 |
|  |  | (b) Indicate number of participants with missing data for each variable of interest | Results paragraph 1, Tables 1 and 2 |
|  |  | (c) Summarise follow-up time (eg, average and total amount) | Results paragraph 1 |
| Outcome data | 15* | Report numbers of outcome events or summary measures over time | Results subsection Characterization of 3GCR-EC Isolates, Fig 3 |

| Main results | 16 | (*a*) Give unadjusted estimates and, if applicable, confounder-adjusted estimates and their precision (eg, 95% confidence interval). Make clear which confounders were adjusted for and why they were included | Results subsections Risk Factors for 3GCR-EC and Risk Factors for ESBL-EC, Table 3 |
| --- | --- | --- | --- |
|  |  | (*b*) Report category boundaries when continuous variables were categorized |  |
|  |  | (*c*) If relevant, consider translating estimates of relative risk into absolute risk for a meaningful time period |  |
| Other analyses | 17 | Report other analyses done—eg analyses of subgroups and interactions, and sensitivity analyses | Results subsections Risk Factors for 3GCR-EC and Risk Factors for ESBL-EC, Tables 4 and 5 |
| **Discussion** | | | |
| Key results | 18 | Summarise key results with reference to study objectives | Discussion paragraph 1 |
| Limitations | 19 | Discuss limitations of the study, taking into account sources of potential bias or imprecision. Discuss both direction and magnitude of any potential bias | Discussion paragraph 6 |
| Interpretation | 20 | Give a cautious overall interpretation of results considering objectives, limitations, multiplicity of analyses, results from similar studies, and other relevant evidence | Discussion final paragraph |
| Generalisability | 21 | Discuss the generalisability (external validity) of the study results | Discussion final paragraph |
| **Other information** | | | |
| Funding | 22 | Give the source of funding and the role of the funders for the present study and, if applicable, for the original study on which the present article is based | See funding statement |

####

**S1 Sample Size & Power Calculations.** This study originally aimed to enroll 360 participants from different households. Using stratified random sampling, we enrolled households in three neighborhood types: 1) with no food-animal production; 2) with small-scale food-animal production only; and 3) with small-scale and commercial-scale food-animal production. The study was, therefore, powered to detect expected differences in prevalence of multidrug- and cephalosporin-resistant (MDR) *E. coli* between neighborhoods of different types. For a comparison between neighborhoods with no food-animal production and those with only small-scale or “backyard” food-animals, assuming an intra-class correlation (ICC) of 0.5, assuming a previous estimate of 13% prevalence of MDR *E. coli* in unexposed neighborhoods, and assuming an alpha of 0.05, the study has 96% power to detect a 2-fold difference in prevalence in neighborhoods with only small-scale food-animal production compared to unexposed neighborhoods. These prevalence and magnitude of effect estimates were assumed based on our previous work and that of Braykov et al. (2016) in Ecuador. For neighborhoods with both exposures to small-scale and commercial-scale food-animal production, where the prevalence of MDR *E. coli* was expected to be even higher at 55%, we would have 100% power to detect this 2-fold difference in prevalence compared to unexposed neighborhoods. Even with an ICC of 0.8, the power is 86% and 100%, respectively.

#### Table A. Prevalence of third-generation cephalosporin-resistant, extended-spectrum beta-lactamase, multidrug-resistant, and extensively drug-resistant *E. coli* among children.

|  | **Data Collection Cycle**  ***n* (%)** | | | | |  |
| --- | --- | --- | --- | --- | --- | --- |
|  | 1 | 2 | 3 | 4 | 5 | Total |
| Total child fecal samples | 376 (100) | 361 (100) | 371 (100) | 230 (100) | 361 (100) | 1699 (100) |
| *ESBL-EC+* samples | 44 (12) | 48 (13) | 26 (7) | 37 (16) | 28 (8) | 183 (11) |
| *MDR-EC+* samples | 164 (44) | 151 (42) | 130 (35) | 76 (33) | 53 (15) | 574 (34) |
| *XDR-EC+* samples | 89 (24) | 78 (22) | 63 (17) | 27 (12) | 15 (4) | 272 (16) |
| Total 3GCR-EC isolates | 273 (100) | 234 (100) | 217 (100) | 115 (100) | 71 (100) | 910 (100) |
| *ESBL-EC isolates* | 51 (19) | 52 (22) | 29 (13) | 40 (35) | 28 (39) | 200 (22) |
| *MDR-EC isolates* | 234 (86) | 219 (94) | 188 (87) | 93 (81) | 53 (75) | 787 (86) |
| *XDR-EC isolates* | 118 (43) | 97 (41) | 76 (35) | 29 (25) | 15 (21) | 335 (37) |

#### 3GCR-EC: third-generation cephalosporin-resistant *E. coli.* ESBL-EC: extended-spectrum beta-lactamase producing *E. coli.* MDR-EC: multidrug-resistant (phenotypically resistant to 3 or more drug classes including third-generation cephalosporin) *E. coli.* XDR-EC: extensively drug-resistant (phenotypically resistant to 5 or more drug classes including third-generation cephalosporin) *E. coli.*

####

#### Table B. Proportion of third-generation cephalosporin-resistant *E. coli* (3GCR-EC) isolates resistant to individual antibiotics in phenotypic susceptibility testing by data collection cycle.

| Cycle | 3GCR-EC  Isolates | **3GCR-EC Isolates Resistant to Antibiotic**  ***n* (%)** | | | | | | | | | |
| --- | --- | --- | --- | --- | --- | --- | --- | --- | --- | --- | --- |
|  |  | AM | CAZ | CIP | CTX | CZ | FEP | GM | IPM | SXT | TE |
| 1 | 273  (100) | 271  (99) | 71  (26) | 136  (50) | 252  (92) | 273  (100) | 119  (44) | 46  (17) | 2  (1) | 182  (67) | 201  (74) |
| 2 | 234  (100) | 233  (100) | 57  (24) | 118  (50) | 222  (95) | 233  (100) | 104  (44) | 48  (21) | 1  (0) | 153  (65) | 182  (78) |
| 3 | 217  (100) | 215  (99) | 48  (22) | 100  (46) | 199  (92) | 215  (99) | 54  (25) | 32  (15) | 1  (0) | 144  (66) | 152  (70) |
| 4 | 115  (100) | 118  (100) | 28  (24) | 42  (36) | 107  (91) | 118  (100) | 33  (28) | 16  (14) | 0  (0) | 61  (52) | 78  (66) |
| 5 | 71  (100) | 67  (94) | 11  (15) | 21  (30) | 58  (82) | 68  (96) | 20  (28) | 8  (11) | 1  (1) | 39  (55) | 40  (56) |
| Total | 910  (100) | 904  (99) | 215  (24) | 417  (46) | 838  (92) | 907  (100) | 330  (36) | 150  (16) | 5  (1) | 579  (64) | 653  (72) |

3GCR-EC: third-generation cephalosporin-resistant *E. coli;* AM: ampicillin; CAZ: ceftazidime; CIP: ciprofloxacin; CTX: cefotaxime; CZ: cefazolin; FEP: cefepime; GM: gentamicin; IPM: imipenem; SXT: trimethoprim/sulfamethoxazole; TE: tetracycline.

####

#### Table C. Phenotypic antibiotic resistance of 3GCR-EC isolates from animal fecal samples (one colony isolated per fecal sample) collected at the same households as child fecal samples, stratified by animal species.

| Species | HH  (S) | 3GCR-EC Isolates | **3GCR-EC Isolates Resistant to Antibiotics**  ***n* (%)** | | | | | | | | | | | |
| --- | --- | --- | --- | --- | --- | --- | --- | --- | --- | --- | --- | --- | --- | --- |
|  |  |  | ESBL | AM | CAZ | CIP | CTX | CZ | FEP | GM | IPM | SXT | TE |  |
| Chickens | 181  (364) | 255  (100) | 53  (21) | 255 (100) | 65 (26) | 109 (43) | 247 (97) | 254 (99) | 107 (42) | 44 (17) | 1  (<1) | 161 (63) | 205 (80) |  |
| Dogs | 354 (903) | 621  (100) | 145 (23) | 616 (99) | 145 (23) | 307 (49) | 598 (96) | 619 (99) | 245 (39) | 128 (21) | 1  (<1) | 417 (67) | 503 (81) |  |
| Pigs | 55 (366) | 80  (100) | 22  (28) | 80 (100) | 29 (36) | 34 (43) | 75 (94) | 80 (100) | 35 (44) | 17 (21) | 0  (0) | 47 (59) | 62 (78) |  |
| Water-fowl/ pheasants* | 81 (101) | 67  (100) | 15  (22) | 67 (100) | 15 (22) | 14 (21) | 67 (100) | 67 (100) | 32 (48) | 11 (16) | 0  (0) | 39 (58) | 61 (91) |  |
| Other**^†^** | 127 (138) | 38  (100) | 5  (13) | 38 (100) | 7  (18) | 7  (18) | 37 (97) | 38 (100) | 15 (39) | 6  (16) | 0  (0) | 20 (53) | 24 (63) |  |
| Total | 376 | 1,191  (100) | 240  (20) | 1,056 (89) | 261  (22) | 471  (40) | 1,024 (86) | 1,058 (89) | 434  (36) | 206  (17) | 2  (<1) | 684  (57) | 855  (72) |  |

#### *Waterfowl/pheasants include ducks, geese, and quail. ^†^Other includes cows, goats, sheep, horses, llamas, guinea pigs, cats, rabbits, and other birds. HH: households; S: stool samples; 3GCR-EC: third-generation cephalosporin-resistant *E. coli*; ESBL: extended-spectrum beta-lactamase producing *E. coli*; AM: ampicillin; CAZ: ceftazidime; CIP: ciprofloxacin; CTX: cefotaxime; CZ: cefazolin; FEP: cefepime; GM: gentamicin; IPM: imipenem; SXT: trimethoprim/sulfamethoxazole; TE: tetracycline.

####

#### Table D. Prevalence of clinically important sequence types (ST) among sequenced 3GCR-EC isolates (N=571) from child fecal samples.

| **ST No. Isolates (%)** |
| --- |
| 10 41 (7.18)  38 27 (4.73)  354 23 (4.03)  131 20 (3.50)  117 19 (3.33)  69 9 (1.58)  23 7 (1.23)  617 7 (1.23)  90 7 (1.23)  58 6 (1.05)  224 5 (0.88)  1193 4 (0.70)  1196 3 (0.53)  457 3 (0.53)  167 2 (0.35)  410 1 (0.18)  88 1 (0.18)  95 1 (0.18) |

####

#### Table E. Proportion of 3GCR-EC isolates with beta-lactamase genes (among 15 most prevalent) detected in whole-genome sequences, stratified by phenotypic ESBL production.

|  | **ESBL- (n=426)** | | | **ESBL+ (n=145)** | | |
| --- | --- | --- | --- | --- | --- | --- |
| Rank | Gene | *n* | *(%)* | Gene | *n* | *(%)* |
| 1 | *bla*_TEM-141_ | 100 | (23.47) | *bla*_CTX-M-55_ | 47 | (32.41) |
| 2 | *bla*_CTX-M-55_ | 83 | (19.48) | *bla*_TEM-141_ | 31 | (21.38) |
| 3 | *bla*_TEM-1B_ | 72 | (16.9) | *bla*_TEM-1B_ | 23 | (15.86) |
| 4 | *bla*_CTX-M-65_ | 50 | (11.74) | *bla*_CTX-M-15_ | 21 | (14.48) |
| 5 | *bla*_CTX-M-15_ | 49 | (11.5) | *bla*_OXA-1_ | 13 | (8.97) |
| 6 | *bla*_TEM-104_ | 44 | (10.33) | *bla*_TEM-102_ | 11 | (7.59) |
| 7 | *bla*_CMY-2_ | 41 | (9.62) | *bla*_CTX-M-65_ | 10 | (6.9) |
| 8 | *bla*_TEM-102_ | 36 | (8.45) | *bla*_TEM-105_ | 10 | (6.9) |
| 9 | *bla*_CTX-M-3_ | 31 | (7.28) | *bla*_SHV-12_ | 9 | (6.21) |
| 10 | *bla*_TEM-105_ | 29 | (6.81) | *bla*_TEM-104_ | 9 | (6.21) |
| 11 | *bla*_CTX-M-27_ | 17 | (3.99) | *bla*_CTX-M-101_ | 7 | (4.83) |
| 12 | *bla*_CTX-M-8_ | 12 | (2.82) | *bla*_CTX-M-27_ | 5 | (3.45) |
| 13 | *bla*_OXA-1_ | 11 | (2.58) | *bla*_SHV-5_ | 4 | (2.76) |
| 14 | *bla*_TEM-176_ | 11 | (2.58) | *bla*_TEM-1A_ | 4 | (2.76) |
| 15 | *bla*_CTX-M-14b_ | 10 | (2.35) | *bla*_CTX-M-14b_ | 3 | (2.07) |

#### 3GCR-EC: third-generation cephalosporin-resistant *E. coli.* ESBL-EC: extended-spectrum beta-lactamase producing *E. coli*

#### Table F. Prevalence of beta-lactamase resistance genes among sequenced 3GCR-EC isolates from children, stratified by parishes and intensity of commercial food animal production.

####

|  |  | **CMY** | | **CTX-M** | | **OXA** | | **SHV** | | **TEM** | |
| --- | --- | --- | --- | --- | --- | --- | --- | --- | --- | --- | --- |
| Parish | CFOs | *n* | *(%)* | *n* | *(%)* | *n* | *(%)* | *n* | *(%)* | *n* | *(%)* |
| **Low-intensity** |  |  |  |  |  |  |  |  |  |  |  |
| Tababela  (n=13) | 2 | 1 | (7.69) | 8 | (61.54) | 4 | (30.77) | 0 | (0.00) | 3 | (23.08) |
| Tumbaco  (n=39) | 8 | 2 | (5.13) | 27 | (69.23) | 3 | (7.69) | 1 | (2.56) | 23 | (58.97) |
| Checa (Chilpa)  (n=84) | 6 | 7 | (8.33) | 57 | (67.86) | 3 | (3.57) | 5 | (5.95) | 50 | (59.52) |
| Pifo  (n=129) | 8 | 9 | (6.98) | 78 | (60.47) | 4 | (3.10) | 4 | (3.10) | 56 | (43.41) |
| **High-intensity** |  |  |  |  |  |  |  |  |  |  |  |
| El Quinche  (n=30) | 32 | 3 | (10.00) | 24 | (80.00) | 0 | (0.00) | 1 | (3.33) | 20 | (66.67) |
| Puembo  (n=39) | 36 | 0 | (0.00) | 29 | (74.36) | 2 | (5.13) | 1 | (2.56) | 19 | (48.72) |
| Yaruqui  (n=234) | 35 | 26 | (11.11) | 143 | (61.11) | 12 | (5.13) | 14 | (5.98) | 122 | (52.14) |

#### CFOs: commercial food animal operations.

####

#### Table G. Average number of total antibiotic resistance genes (ARGs) per third-generation cephalosporin-resistant *E. coli* isolate from children, stratified by parish.

|  |  | **Total ARGs** | |
| --- | --- | --- | --- |
| Parish | CFOs | Mean | (SD) |
| **Low-intensity** |  |  |  |
| Tababela  (n=13) | 2 | 10.78 | (2.49) |
| Tumbaco  (n=39) | 8 | 10.48 | (4.93) |
| Checa (Chilpa) (n=84) | 6 | 10.01 | (4.70) |
| Pifo  (n=129) | 8 | 9.52 | (4.12) |
| **High-intensity** |  |  |  |
| El Quinche  (n=30) | 32 | 8.29 | (4.66) |
| Puembo  (n=39) | 36 | 10.07 | (4.21) |
| Yaruqui  (n=234) | 35 | 9.66 | (4.25) |

#### CFOs: commercial food animal operations.

####

#### Table H. Prevalence of CTX-M-type genes among sequenced 3GCR-EC isolates from children, stratified by parish and intensity of commercial food animal production.

####

|  | **Low-intensity** | | | | | | | | **High-intensity** | | | | | |
| --- | --- | --- | --- | --- | --- | --- | --- | --- | --- | --- | --- | --- | --- | --- |
|  | Tababela (n=13) | | Tumbaco (n=39) | | Checa (Chilpa) (n=84) | | Pifo  (n=129) | | El Quinche (n=30) | | Puembo (n=39) | | Yaruqui (n=234) | |
|  | *n* | *%* | *n* | *%* | *n* | *%* | *n* | *%* | *n* | *%* | *n* | *%* | *n* | *%* |
| *bla*_CTX-M-55_ | 2 | 15.38 | 12 | 30.77 | 18 | 21.43 | 27 | 20.93 | 4 | 13.33 | 12 | 30.77 | 55 | 23.50 |
| *bla*_CTX-M-65_ | 2 | 15.38 | 3 | 7.69 | 10 | 11.90 | 13 | 10.08 | 2 | 6.67 | 4 | 10.26 | 25 | 10.68 |
| *bla*_CTX-M-15_ | 4 | 30.77 | 4 | 10.26 | 8 | 9.52 | 14 | 10.85 | 13 | 43.33 | 6 | 15.38 | 21 | 8.97 |
| *bla*_CTX-M-27_ | 0 | 0.00 | 3 | 7.69 | 1 | 1.19 | 5 | 3.88 | 1 | 3.33 | 1 | 2.56 | 11 | 4.70 |
| *bla*_CTX-M-3_ | 0 | 0.00 | 3 | 7.69 | 9 | 10.71 | 9 | 6.98 | 0 | 0.00 | 0 | 0.00 | 11 | 4.70 |
| *bla*_CTX-M-14b_ | 0 | 0.00 | 0 | 0.00 | 5 | 5.95 | 0 | 0.00 | 0 | 0.00 | 1 | 2.56 | 7 | 2.99 |
| *bla*_CTX-M-101_ | 0 | 0.00 | 0 | 0.00 | 2 | 2.38 | 2 | 1.55 | 0 | 0.00 | 0 | 0.00 | 5 | 2.14 |
| *bla*_CTX-M-8_ | 0 | 0.00 | 1 | 2.56 | 3 | 3.57 | 0 | 0.00 | 3 | 10.00 | 1 | 2.56 | 5 | 2.14 |
| *bla*_CTX-M-103_ | 0 | 0.00 | 0 | 0.00 | 0 | 0.00 | 0 | 0.00 | 0 | 0.00 | 0 | 0.00 | 1 | 0.43 |
| *bla*_CTX-M-12_ | 0 | 0.00 | 0 | 0.00 | 0 | 0.00 | 0 | 0.00 | 0 | 0.00 | 0 | 0.00 | 1 | 0.43 |
| *bla*_CTX-M-124_ | 0 | 0.00 | 0 | 0.00 | 0 | 0.00 | 0 | 0.00 | 0 | 0.00 | 0 | 0.00 | 1 | 0.43 |
| *bla*_CTX-M-130_ | 0 | 0.00 | 0 | 0.00 | 0 | 0.00 | 0 | 0.00 | 0 | 0.00 | 0 | 0.00 | 1 | 0.43 |
| *bla*_CTX-M-14_ | 0 | 0.00 | 1 | 2.56 | 1 | 1.19 | 2 | 1.55 | 2 | 6.67 | 2 | 5.13 | 1 | 0.43 |
| *bla*_CTX-M-148_ | 0 | 0.00 | 0 | 0.00 | 0 | 0.00 | 0 | 0.00 | 0 | 0.00 | 0 | 0.00 | 1 | 0.43 |
| *bla*_CTX-M-162_ | 0 | 0.00 | 0 | 0.00 | 0 | 0.00 | 1 | 0.78 | 0 | 0.00 | 0 | 0.00 | 1 | 0.43 |
| *bla*_CTX-M-5_ | 0 | 0.00 | 0 | 0.00 | 0 | 0.00 | 0 | 0.00 | 0 | 0.00 | 0 | 0.00 | 1 | 0.43 |
| *bla*_CTX-M-64_ | 0 | 0.00 | 0 | 0.00 | 0 | 0.00 | 0 | 0.00 | 0 | 0.00 | 1 | 2.56 | 1 | 0.43 |
| *bla*_CTX-M-123_ | 0 | 0.00 | 0 | 0.00 | 0 | 0.00 | 2 | 1.55 | 0 | 0.00 | 1 | 2.56 | 0 | 0.00 |
| *bla*_CTX-M-10_ | 0 | 0.00 | 0 | 0.00 | 0 | 0.00 | 1 | 0.78 | 0 | 0.00 | 0 | 0.00 | 0 | 0.00 |
| *bla*_CTX-M-114_ | 0 | 0.00 | 0 | 0.00 | 0 | 0.00 | 1 | 0.78 | 0 | 0.00 | 0 | 0.00 | 0 | 0.00 |
| *bla*_CTX-M-144_ | 0 | 0.00 | 0 | 0.00 | 0 | 0.00 | 1 | 0.78 | 0 | 0.00 | 0 | 0.00 | 0 | 0.00 |
| *bla*_CTX-M-2_ | 0 | 0.00 | 0 | 0.00 | 1 | 1.19 | 1 | 0.78 | 0 | 0.00 | 0 | 0.00 | 0 | 0.00 |
| *bla*_CTX-M-164_ | 0 | 0.00 | 0 | 0.00 | 1 | 1.19 | 0 | 0.00 | 0 | 0.00 | 0 | 0.00 | 0 | 0.00 |
| *bla*_CTX-M-179_ | 0 | 0.00 | 0 | 0.00 | 1 | 1.19 | 0 | 0.00 | 0 | 0.00 | 0 | 0.00 | 0 | 0.00 |

####

####

#### Table I. Sensitivity analysis results for main analysis associations between combined food-animal exposures and 3GCR-EC and ESBL-EC including only one isolate per child fecal sample.

|  |  | Adjusted RR for 3GCR-EC  (95% CI) | | Adjusted RR for ESBL-EC  (95% CI) | |
| --- | --- | --- | --- | --- | --- |
|  |  | No household food animals | Household  food animals | No household food animals | Household  food animals |
| **Commercial Food Animal Operations in 5 km Radius** | ≤ 5 | 1.00 (ref) | 1.25 (0.97, 1.61) | 1.00 (ref) | 1.17 (0.65, 2.11) |
|  | > 5 | 1.30 (1.10, 1.53) | 1.26 (1.04, 1.53) | 1.10 (0.74, 1.64) | 1.07 (0.67, 1.70) |
|  | ≥ 5 within strata of household food animals | 1.30 (1.10, 1.53) | 1.01 (0.79, 1.29) | 1.10 (0.74, 1.64) | 0.92 (0.49, 1.70) |
| **Distance to Nearest Commercial Operation** | ≥ 1.5 km | 1.00 (ref) | 1.07 (0.86, 1.35) | 1.00 (ref) | 0.78 (0.44, 1.39) |
|  | < 1.5 km | 1.14 (0.97, 1.33) | 1.21 (1.02, 1.45) | 0.90 (0.61, 1.32) | 1.13 (0.73, 1.77) |
|  | < 1.5 km within strata of household food animals | 1.14 (0.97, 1.33) | 1.13 (0.90, 1.42) | 0.90 (0.61, 1.32) | 1.45 (0.79, 2.64) |
| **Distance to Commercial Operation Drainage Path** | > 500 m | 1.00 (ref) | 0.98 (0.79, 1.23) | 1.00 (ref) | 0.83 (0.45, 1.52) |
|  | 101-500 m | 0.98 (0.82, 1.16) | 1.16 (0.89, 1.52) | 1.07 (0.71, 1.6) | 1.23 (0.61, 2.49) |
|  | ≤ 100 m | 1.05 (0.84, 1.31) | 1.10 (0.85, 1.43) | 0.77 (0.40, 1.49) | 1.76 (0.83, 3.72) |
|  | 101-500 m within strata of household food animals | 0.98 (0.82, 1.16) | 1.15 (0.92, 1.42) | 1.07 (0.71, 1.6) | 1.02 (0.61, 1.72) |
|  | ≤ 100 m within strata of household food animals | 1.05 (0.84, 1.31) | 1.08 (0.87, 1.34) | 0.77 (0.40, 1.49) | 1.07 (0.67, 1.70) |

Adjusted RRs and robust 95% CIs estimated using log-binomial regression models with generalized estimating equations included interaction terms between commercial and household food animal exposure variables, and included the following covariates: caregiver education, asset score, child age and sex, and child antibiotic use in the last 3 months. N=1,677 observations across 1,677 child fecal samples (including 663 total 3GCR-EC isolates) for 594 children (number of observations includes multiple isolates per fecal sample). RR: Relative Risk. CI: 95% confidence interval. 3GCR-EC: third-generation cephalosporin-resistant *E. coli.* ESBL: extended-spectrum beta-lactamase producing *E. coli*.

#### Table J. Associations between secondary risk factors and 3GCR-EC carriage among children.

| Risk Factor | N | Unadjusted RR  (95% CI) | *P*-value | Adjusted RR  (95% CI) | *P*-value |
| --- | --- | --- | --- | --- | --- |
| Household animals received antibiotics in last 6 months (vs. not) | 1922 | 1.22 (1.01, 1.47) | 0.036 | 1.15 (0.95, 1.39) | 0.163 |
| Livestock/poultry drank irrigation water in last 3 weeks (vs. not) | 955 | 1.22 (0.94, 1.57) | 0.136 | 1.21 (0.94, 1.56) | 0.140 |
| Animal feces left in yard  (vs. placed in trash) | 1133 | 0.88 (0.73, 1.07) | 0.192 | 0.88 (0.73, 1.07) | 0.201 |
| Animal feces placed on land/crops  (vs. placed in trash) | 1133 | 1.06 (0.88, 1.28) | 0.530 | 1.04 (0.86, 1.26) | 0.676 |
| Household owns dogs  (vs. none) | 1949 | 1.02 (0.90, 1.15) | 0.778 | 1.02 (0.90, 1.15) | 0.783 |
| Household owns chickens  (vs. none) | 1949 | 1.12 (0.99, 1.26) | 0.067 | 1.10 (0.98, 1.24) | 0.104 |
| Household owns pigs  (vs. none) | 1948 | 1.28 (1.08, 1.52) | 0.006 | 1.23 (1.02, 1.48) | 0.030 |
| 3GCR-EC+ animal feces in yard  (vs. ESBL-EC- feces) | 1144 | 1.04 (0.89, 1.20) | 0.645 | 1.03 (0.90, 1.19) | 0.657 |
| ESBL-EC+ animal feces in yard  (vs. 3GCR-EC- feces) | 1144 | 1.09 (0.93, 1.30) | 0.295 | 1.11 (0.95, 1.30) | 0.188 |
| Caregiver worked with animals in last 6 months^a^ (vs. not) | 1949 | 0.93 (0.80, 1.08) | 0.334 | 0.95 (0.82, 1.10) | 0.485 |
| Child contact with livestock in last  3 months (vs. not) | 1949 | 1.00 (0.88, 1.13) | 0.955 | 1.04 (0.92, 1.17) | 0.552 |
| Child contact with pets in last  3 months (vs. not) | 1949 | 1.17 (1.03, 1.33) | 0.015 | 1.23 (1.09, 1.39) | 0.001 |
| Child played near animal feces in last  3 weeks (vs. not) | 1942 | 0.92 (0.82, 1.04) | 0.190 | 0.99 (0.88, 1.12) | 0.917 |
| Child rarely/never handwashes after contact with animals  (vs. sometimes/always) | 1882 | 1.20 (1.01, 1.42) | 0.035 | 1.15 (0.98, 1.34) | 0.087 |

All RRs and robust 95% CIs estimated using log-binomial regression models with generalized estimating equations to adjust for repeated measures. Models for adjusted RRs included the following covariates: caregiver education, asset score, child age and sex, and child antibiotic use in the last 3 months. RR: relative risk. CI: confidence interval.

^a^ Includes working with live animals, animal feces, or meat processing.

#### Table K. Associations between secondary risk factors and ESBL-EC carriage among children.

| Risk Factor | N | Unadjusted RR  (95% CI) | *P*-value | Adjusted RR  (95% CI) | *P*-value |
| --- | --- | --- | --- | --- | --- |
| Household animals received antibiotics in last 6 months (vs. not) | 1922 | 1.23 (0.68, 2.21) | 0.492 | 1.15 (0.63, 2.11) | 0.655 |
| Livestock/poultry drank irrigation water in last 3 weeks (vs. not) | 955 | 1.19 (0.70, 2.01) | 0.517 | 1.21 (0.72, 2.04) | 0.464 |
| Animal feces left in yard  (vs. placed in trash) | 1133 | 0.69 (0.41, 1.15) | 0.154 | 0.69 (0.40, 1.16) | 0.162 |
| Animal feces placed on land/crops  (vs. placed in trash) | 1133 | 1.59 (1.08, 2.33) | 0.019 | 1.63 (1.09, 2.46) | 0.019 |
| Household owns dogs  (vs. none) | 1949 | 1.36 (1.01, 1.84) | 0.045 | 1.35 (1.00, 1.83) | 0.053 |
| Household owns chickens  (vs. none) | 1949 | 1.17 (0.85, 1.61) | 0.341 | 1.16 (0.84, 1.61) | 0.361 |
| Household owns pigs  (vs. none) | 1948 | 1.51 (0.96, 2.36) | 0.073 | 1.46 (0.93, 2.28) | 0.097 |
| 3GCR-EC+ animal feces in yard  (vs. ESBL-EC- feces) | 1144 | 1.10 (0.80, 1.51) | 0.553 | 1.10 (0.80, 1.51) | 0.575 |
| ESBL-EC+ animal feces in yard  (vs. 3GCR-EC- feces) | 1144 | 1.40 (0.96, 2.03) | 0.077 | 1.39 (0.96, 2.02) | 0.082 |
| Caregiver worked with animals in last 6 months^a^ (vs. not) | 1949 | 1.14 (0.82, 1.60) | 0.435 | 1.13 (0.81, 1.60) | 0.474 |
| Child contact with livestock in last  3 months (vs. not) | 1949 | 1.01 (0.76, 1.36) | 0.929 | 1.02 (0.76, 1.38) | 0.880 |
| Child contact with pets in last  3 months (vs. not) | 1949 | 1.51 (1.08, 2.11) | 0.016 | 1.54 (1.10, 2.16) | 0.012 |
| Child played near animal feces in last  3 weeks (vs. not) | 1942 | 1.07 (0.80, 1.43) | 0.648 | 1.09 (0.81, 1.47) | 0.568 |
| Child rarely/never handwashes after contact with animals  (vs. sometimes/always) | 1882 | 1.148 (0.74, 1.79) | 0.544 | 1.14 (0.73, 1.78) | 0.570 |

All RRs and robust 95% CIs estimated using log-binomial regression models with generalized estimating equations to adjust for repeated measures. Models for adjusted RRs included the following covariates: caregiver education, asset score, child age and sex, and child antibiotic use in the last 3 months. RR: relative risk. CI: confidence interval.

^a^ Includes working with live animals, animal feces, or meat processing.

#### Table L. Secular trends in caregiver-reported child illness and antibiotic use stratified by household food animal ownership.

|  | **Data Collection Cycle** | | | | |
| --- | --- | --- | --- | --- | --- |
|  | 1  *n* (%) | 2  *n* (%) | 3  *n* (%) | 4  *n* (%) | 5  *n* (%) |
| **No household food animals** |  |  |  |  |  |
| *Child had diarrhea in last 7 days* | 48 (18.6) | 46 (19.2) | 28 (10.9) | 25 (15.4) | 11 (4.2) |
| Missing | 1 (0.4) | 1 (0.4) | 0 (0.0) | 0 (0.0) | 2 (0.8) |
| *Child treated for infection in last 3 months* | 77 (29.8) | 69 (28.7) | 66 (25.6) | 42 (25.9) | 21 (8.0) |
| Missing | 2 (0.8) | 1 (0.4) | 0 (0.0) | 0 (0.0) | 0 (0.0) |
| *Child took antibiotics in last 3 months* | 62 (24.0) | 46 (19.2) | 48 (18.6) | 18 (11.1) | 15 (5.7) |
| Missing | 0 (0.0) | 1 (0.4) | 0 (0.0) | 0 (0.0) | 0 (0.0) |
| **Household food animals** |  |  |  |  |  |
| *Child had diarrhea in last 7 days* | 27 (24.5) | 31 (26.5) | 15 (14.0) | 11 (17.5) | 4 (4.3) |
| Missing | 1 (0.9) | 0 (0.0) | 1 (0.9) | 0 (0.0) | 0 (0.0) |
| *Child treated for infection in last 3 months* | 38 (34.5) | 29 (24.8) | 18 (16.8) | 12 (19.0) | 8 (8.7) |
| Missing | 1 (0.9) | 0 (0.0) | 0 (0.0) | 0 (0.0) | 0 (0.0) |
| *Child took antibiotics in last 3 months* | 35 (31.8) | 21 (17.9) | 15 (14.0) | 8 (12.7) | 6 (6.5) |
| Missing | 0 (0.0) | 1 (0.9) | 0 (0.0) | 0 (0.0) | 0 (0.0) |

####

**Table M.** Access to water and sanitation at households included in the main analysis (N=594).

|  | *n* (%) |
| --- | --- |
| Piped drinking water inside home | 548 (92.3) |
| 24-hr drinking water access | 586 (98.7) |
| Flush toilet to sewer or septic | 587 (98.8) |

#### Figure A. Directed acyclic graph of causal relationship between exposures to commercial and household food animal production and ESBL-*E. coli* carriage in children. SES: socioeconomic status. ESBL: extended-spectrum beta-lactamase *E. coli.*

####
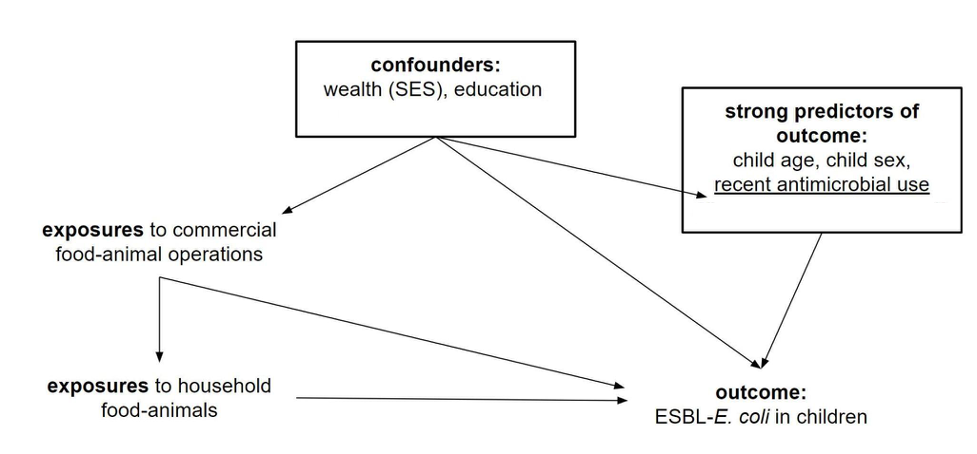


#### Figure B. Flow chart of enrollment and follow-up by data collection cycle. Households were included in the final analysis if they had the necessary exposure, outcome, and covariate data.


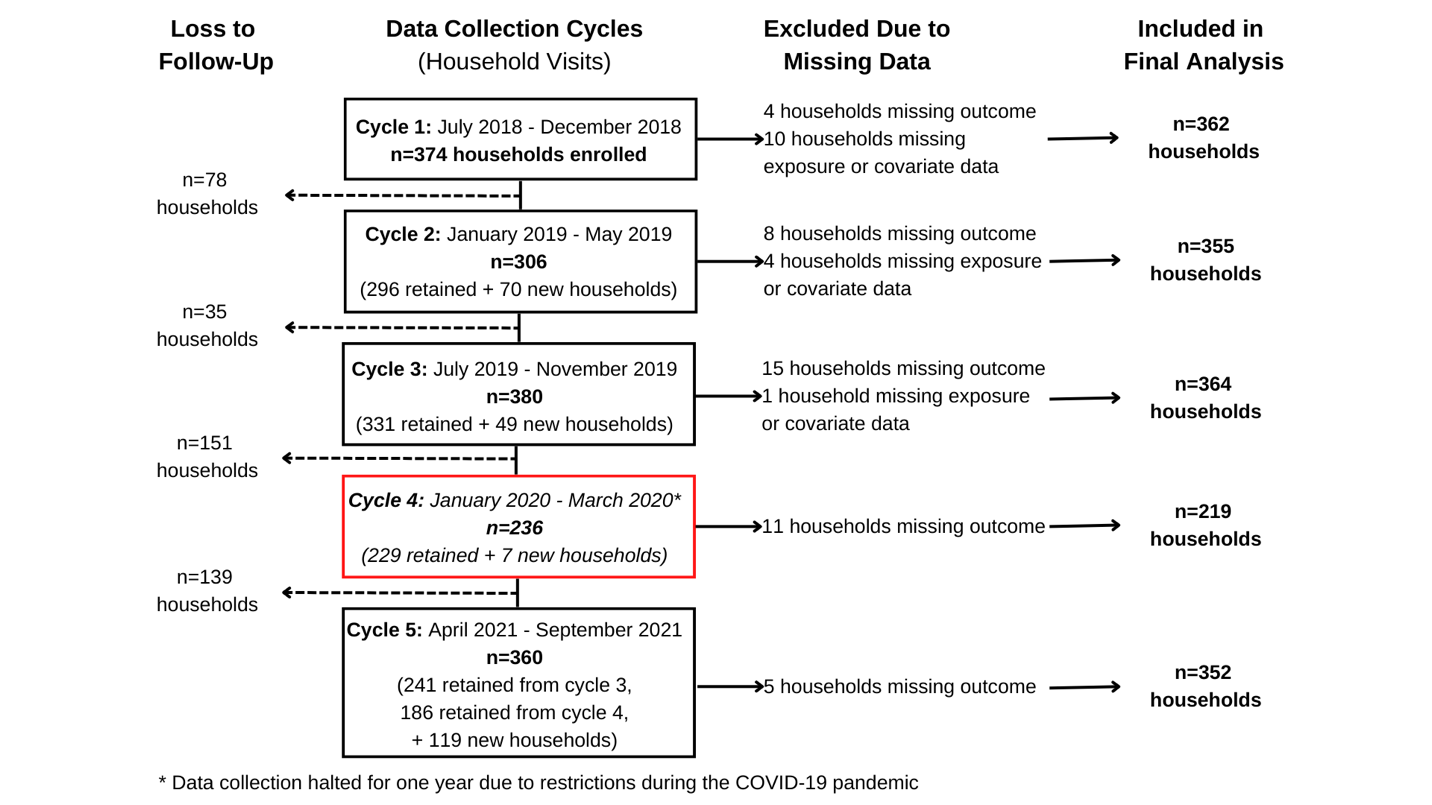


####

#### Figure C. Prevalence of beta-lactamase genes (top 15 most prevalent) among sequenced third-generation cephalosporin-resistant *E. coli* isolates from children, stratified by phenotypic extended-spectrum beta-lactamase (ESBL) production.

####
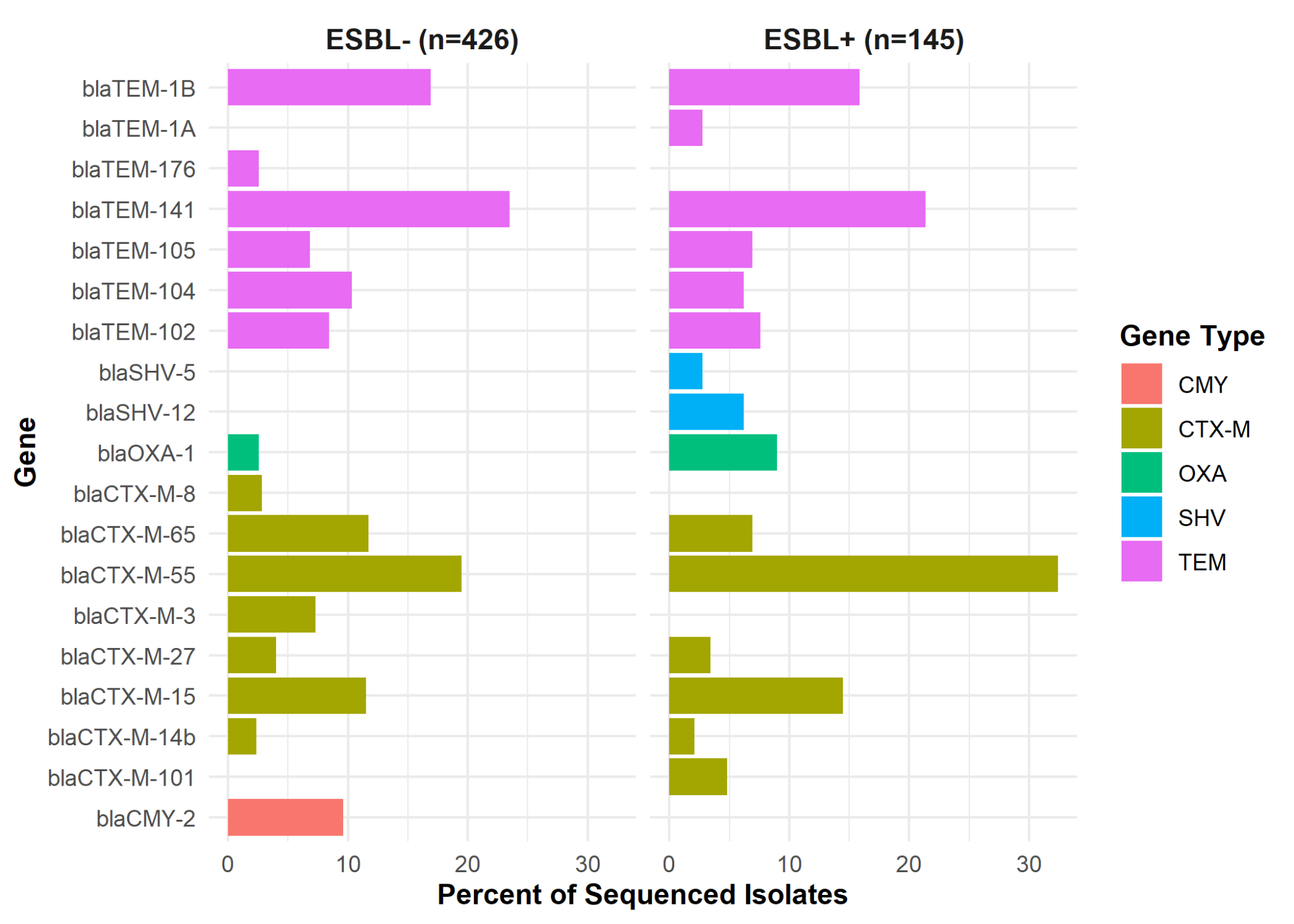


####

### Figure D. Prevalence of beta-lactamase genes by type among third-generation cephalosporin-resistant *E. coli* (3GCR-EC) isolated from children, stratified by parish.

**
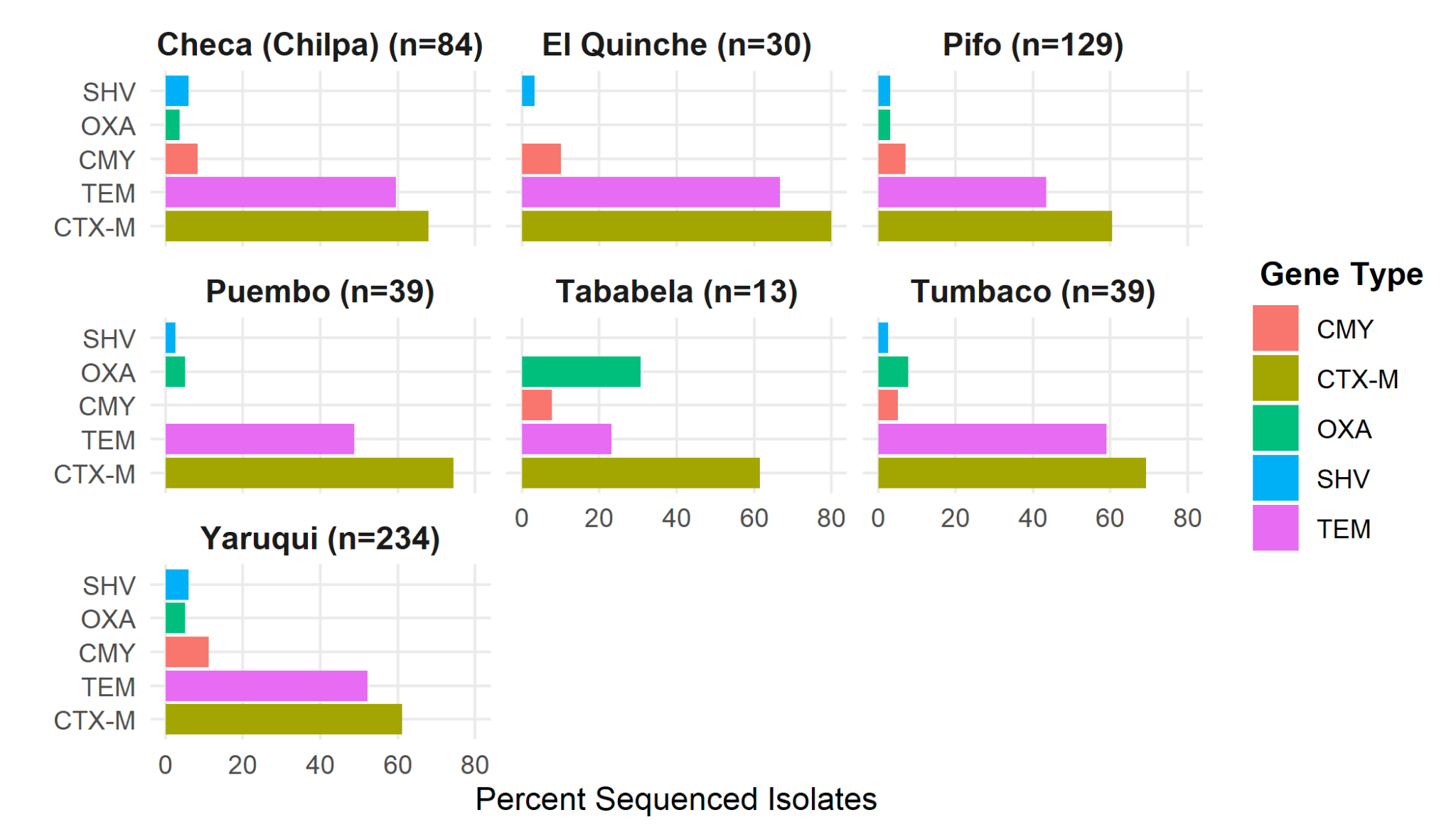
**
