# Supplementary material for: Risk factors for extended-spectrum beta-lactamase (ESBL)-producing E. coli carriage among children in a food animal-producing region of Ecuador: A repeated measures observational study
Source: PLoS Med. 2023 Oct 13;20(10):e1004299. doi: 10.1371/journal.pmed.1004299 (PMC10621961; doi:10.1371/journal.pmed.1004299)
Supplement: Attachment — Submitted filename: amato.pdf [file pmed.1004299.s002.pdf]

This paper discusses the link between food and domestic animals and bacterial carriage in children in Ecuador.

I was asked for a statistical report and I interpret that to include all aspects of the design and conduct of the study.

Points of detail

Page 5 Was there a formal sample size determination to arrive at 360 households? What were the stratification variables?

Page 6 The finding of Amato et al here does not refer to Ecuador does it? Perhaps clarify.

Page 9 There is limited information here about the categorisation of variables. Categorising an essentially continuous variable wastes information (Altman and Royston, 2006; Royston et al., 2006) and leads to models which are often implausible as they predict the effect remaining flat within categories and then jumping to a new value at the category boundary. The position would of course have been worse if the choice had been made with knowledge of the data (Altman et al., 1994).

Page 10 Good to see the packages properly cited.

Page 11 I am not clear about how the final figure of 1940 observations arises. It seems to exceed the number of isolates mentioned earlier in the paragraph.

Page 12 Elsewhere in the article they are called pigs so does the use of hog here imply something? Are they distinct animals in US English as in my dialect we usually only use hog to mean someone or something which consumes excessive resources?

Page 12 I wonder whether means and standard deviations are best for such skewed variables. Perhaps medians and quartiles would be more representative.

Page 14 and 15 I found some of these hard to grasp. First of all it is not always clear whether the effects, particularly the interactions, are significant. As a separate point it was only after some thought that I decided that in Table 3 strata probably means stratum. I do not have a problem with the analysis but some clarification here might be helpful.

Page 19 It is obviously undesirable, although perfectly understandable, that there was major drop–out but what exactly does the use of non–

parametric measures mean and how are they supposed to have dealt with possible selection effects?

Page 20 The phrase 'are needed in LMICs' implies the authors think upper–income countries are blameless. I wish I could be so confident about my own country.

Figure 1 What does the inset show us? Is it possible to superimpose the parish boundaries as it is hard to read this one and Figure 2 together?

Figure 2 This is one of the few places where pie charts might be useful.

Figure 3 We could use a bit more explanation somewhere about what notations like C 500m + HH* mean.

Supplementary figure S1 This is an excellent idea to show us the underlying theory but household members usage of antibiotics does not seem to be among the risk factors presented earlier (in Figure 4 for instance).

Points of more substance

Design

I am not clear about how the design maps on to the scientific and public health question.

- Why was it decided to use a repeated measures design when secular effects seem to be of limited importance? It would have been simpler to sample five times as many households once. Secular effects are mentioned on pages 11 and 12but the discussion on page 17 is couched in terms of COVID–19 which can hardly have been an issue at the design stage.
- Why choose these contiguous parishes? Would there not have been more variety if they were more separated?
- It is hard to find information about the parishes but the sample sizes mentioned in Table 1 do not appear to bear any obvious relationship to the populations mentioned in the Wikipedia articles (of those which have such an article) linked from `https://es.wikipedia.org/wiki/Distrito_Metropolitano_de_Quito`.

Incidentally when I read the title I was confused as I did not expect a major South American city to have food production. It does become clearer in the text that the parishes are within the canton of Quito or the metropolitan region not Quito cityitself.

Context and generalisability

After reading this I do not feel I have much grasp on the way of life of these people and their animal husbandry. This would be important for people to assess generalisability to other regions of the world. Is this a region of subsistence farmers or do most householders have work outside the household and supplement their diet it with home–grown produce? Are they raising the animals for meat or for eggs or milk since given which end of the animal eggs and milk come from the risks of *E coli* must be different than from meat.

Dogs

Much of the article refers to food animals but Supplementary Table S3 shows that the predominant species for stool samples was dogs which I assume are companion animals or working dogs not a food source. What role do dogs play in the transmission?

Summary

Given my background in epidemiology it is perhaps not surprising that I found this rather lacking in detail about the human beings or the animals compared with the detail on bacteria.

Michael Dewey

References

D G Altman and P Royston. The cost of dichotomising continuous variables. *British Medical Journal*, 332:1080, 2006.

D G Altman, B Lausen, W Sauerbrei, and M Schumacher. Dangers of using 'optimal' cutpoints in the evaluation of prognostic factors. *Journal of the National Cancer Institute*, 86:829–835, 1994.

P Royston, D G Altman, and W Sauerbrei. Dichotomizing continuous predictors in multiple regression: a bad idea. *Statistics in Medicine*, 25:127–141, 2006.
