# Supplementary material for: Risk factors for extended-spectrum beta-lactamase (ESBL)-producing E. coli carriage among children in a food animal-producing region of Ecuador: A repeated measures observational study
Source: PLoS Med. 2023 Oct 13;20(10):e1004299. doi: 10.1371/journal.pmed.1004299 (PMC10621961; doi:10.1371/journal.pmed.1004299)
Supplement: Attachment — Submitted filename: Reviewers comments R2.docx [file pmed.1004299.s004.docx]

I think the manuscript has improved in terms of length and clarity. However, some of my bigger concerns remain partially. Below, you will find my comments in more detail. I would like to stress that I don’t understand that the authors decided to stick with the analysis on isolate level instead of analyzing on the individual level (as raised by almost all reviewers).

Introduction

I think the introduction has improved in its focus and it is more concise. However, I think the line in the first two paragraphs is still somewhat shaky. I don’t understand some of the choices made in the order of the addressed background information. It switches a few times from ABR to ESBL and back. The same goes for health impact (addressed in both paragraphs). All information is relevant, but can be improved in ordering.

Materials and Methods

In the inclusion criteria you mention the consent of the caregiver as the third criteria. I would say it is a given that you need consent. I would just make a statement expressing that consent was given by the caretakers for al study participants (or any other covering phrase).

In the statistical analyses it is not stated at what level the outcome is (isolate level, which I still disagree on).

You described a sensitivity analysis with only the first isolate. I would say the same should have been done for the prevalence of carriage (since the sensitivity for finding a positive results is higher when taking more parallel observations per individual). Or at least discuss the impact of a different number of isolates between individuals.

Results

This part has changed massively. However, I still am confused sometimes about the numbers. In line 332-334 is stated how many isolates were collected from how many children from how many households. By including the number of isolates where cephalosporin resistant it get’s less clear. I would not put in outcome results mixed with output results.

Line 377 and further: In how many of the children with at least one positive isolates, positive isolates were also found in animals?

Line 388: how were the 200 additional isolates selected?

Line 406: please delete ‘all fecal samples’, because you are modeling at the isolate level.

Line 419: do you mean marginally significant? Instead of marginally significant I would suggest to use the term borderline significant.

If I understood correctly, another manuscript is discussing the similarity between the isolates collected within epidemiologically linked clusters (humans and animals from the same household). The combination of an epi analysis and the isolate characteristics are telling a much fuller view on the matter. I really don’t understand why this information is not used in this manuscript to support the findings.

Discussion

Line 445-449: Please delete, you don’t have to summarize the study.

Line 461: Depending on the ARB/ARG, but most of the studies do not report transmission from humans with intense contact with animals to other humans. You might want to nuance this statement a bit.

469-474: Please delete, I don’t see any reason to state this. It does not interpret results or highlight any finding in a broader context.

Line 543: I don’t agree with your reasoning regarding your choice on what level to perform the analysis. Especially in terms of translating and interpreting the results. A risk factor for a positive isolate is less interesting, since the exposure is present (and therefore also focus for mitigation) on the individual level. And not on the isolate level. I would strongly suggest (as the other reviewers did as well) to model at the individual level and do a sensitivity level as you did with an individual outcome only based on the first isolate. Furthermore, I find the term ‘some slight differences’ rather vague.

Line 546: Please replace 1 by one.

Line 566-594: Although I understand the request for including implications, I find this way too long. I am sure you can describe the points you are raising in a much more concise way.
