# Supplementary material for: Risk factors for extended-spectrum beta-lactamase (ESBL)-producing E. coli carriage among children in a food animal-producing region of Ecuador: A repeated measures observational study
Source: PLoS Med. 2023 Oct 13;20(10):e1004299. doi: 10.1371/journal.pmed.1004299 (PMC10621961; doi:10.1371/journal.pmed.1004299)
Supplement: Attachment — Submitted filename: PLOSMed Response to Round 2 Reviews.docx [file pmed.1004299.s005.docx]

Requests from Editors:

GENERAL
Please respond to all editor and reviewer comments detailed below, in full.

TITLE
Please revise your title according to PLOS Medicine's style. Your title must be nondeclarative and not a question. It should begin with main concept if possible. "Effect of" should be used only if causality can be inferred, i.e., for an RCT. Please place the study design ("A randomized controlled trial," "A retrospective study," "A modelling study," etc.) in the subtitle (ie, after a colon). Please include the country of origin (Ecuador) in the title.

**We have added the study design after a colon; the country of origin is already in the title.**

AUTHOR SUMMARY
Thank you for including an author summary which reads very nicely but is missing some detail. Please see the information under each of the headings below and revise accordingly (specifically, sample sizes and limitations are notably absent in the current version). Please keep in mind that the summary should consist of 2-3 succinct bullet points under each heading:
• Why Was This Study Done? Authors should reflect on what was known about the topic before the research was published and why the research was needed.
• What Did the Researchers Do and Find? Authors should briefly describe the study design that was used and the study’s major findings. Do include the headline numbers from the study, such as the sample size and key findings.
• What Do These Findings Mean? Authors should reflect on the new knowledge generated by the research and the implications for practice, research, policy, or public health. Authors should also consider how the interpretation of the study’s findings may be affected by the study limitations. In the final bullet point of ‘What Do These Findings Mean?’, please describe the main limitations of the study in non-technical language.

**We have reduced the number of bullet points to 3 bullets under each heading.**

INTRODUCTION
Suggest moving lines 110-113 ending ‘…mortality [7–10].’ to paragraph 1, line 98 preceding, ‘Anti-biotic resistant bacteria’
Suggest new paragraph at line 104 sentence beginning, ‘With the rapid…’ and combing with sentence at line 113 beginning asymptomatic carriage.
Line 126 – please use one or the other of the two in-text reference callouts.

Line 134 – please change ‘feces’ to ‘faeces’

**Each of the above changes have been made accordingly.**

METHODS and RESULTS
Please also see statistical reviewer comments (reviewer #4) below.
In reference to reviewer #4 comments (please see below) please clarify how the sample size was determined. - **Apologies for omitting this in the previous response to comments – we have added a thorough explanation of power calculations and sample size for the parent study in the Supplemental Materials. We have also added the following sentence in the Methods under Study Design: “For power calculations and sample size determination, please see Supplemental Materials (S2. Sample Size & Power Calculations).”**
Line 170 – please state if consent was written or oral

**We have specified that consent was written.**

DISCUSSION
Please begin the introduction with a short clear summary of the article’s findings.
Line 512 - please revise to read SARS-CoV-2 also at line 560, please check and amend throughout.

**The first paragraph of the discussion has been revised to remove the methods and only include a short summary of the article’s findings. COVID-19 has been changed throughout to SARS-CoV-2.**

SUPPORTING INFORMATION
Figure S1 – please see statistical reviewer (#4) comments below. – **This figure has been corrected, please see response below.**

REFERENCES
Throughout, for in-text reference callouts, please remove spaces from between citations. For example, line 100 should read ‘[2,3]’ as opposed to ‘[2, 3]’. **Unfortunately it is not possible to change the citation formats because citations are autogenerated using a citation management software. To avoid citation errors, we’d like to avoid manually editing the citations at this stage. Is this a change that could happen during the proofing process?**

SOCIAL MEDIA
If not already done so, to help us extend the reach of your research, please detail any Twitter handles you wish to be included when we tweet this paper (including your own, your coauthors’, your institution, funder, or lab) in the manuscript submission form when you re-submit the manuscript.


Comments from Reviewers:

Reviewer #1: The revised manuscript has improved considerably and all the points I raised have been answered appropriately.

A few additional very minor suggestions from my side:
- Line 139: I would not only say that risks of "antibiotic-resistant and ESBL-E infections" are urgently needed to be quantified, but also include carriage here, since this is what the main objective of the study and this data is also still sparce from LMICs. Adapt "antibiotic-resistant and ESBL-E infections" to "antibiotic-resistant and ESBL-E carriage and infections". I would also consider to include this to line 459 and the conclusion (line 604). – **Thank you for this suggestion! We have added carriage to both of these sentences.**
- Line 126: reference 18 is included twice. **– This reference has been removed.**
- Line 439: change "0.1080" into "0.108". – **This has been corrected.**
- Line 554: "enterobacteriales" should read "Enterobacterales". – **This has been corrected.**


Reviewer #2: I think the manuscript has improved in terms of length and clarity. However, some of my bigger concerns remain partially. Below, you will find my comments in more detail. I would like to stress that I don't understand that the authors decided to stick with the analysis on isolate level instead of analyzing on the individual level (as raised by almost all reviewers).

Introduction
I think the introduction has improved in its focus and it is more concise. However, I think the line in the first two paragraphs is still somewhat shaky. I don't understand some of the choices made in the order of the addressed background information. It switches a few times from ABR to ESBL and back. The same goes for health impact (addressed in both paragraphs). All information is relevant, but can be improved in ordering. – **We have reordered the background information to emphasize the health impact earlier on.**

Materials and Methods
In the inclusion criteria you mention the consent of the caregiver as the third criteria. I would say it is a given that you need consent. I would just make a statement expressing that consent was given by the caretakers for al study participants (or any other covering phrase). – **Thank you for this suggestion. We have separated the statement on consent from inclusion criteria: “Informed written consent was provided by each caretaker prior to participation in the study.”**
In the statistical analyses it is not stated at what level the outcome is (isolate level, which I still disagree on). – **The statistical analysis section clearly states the following: “The outcomes, 3GCR-EC and ESBL-EC carriage, were treated as binary and modelled at the isolate level…”**
You described a sensitivity analysis with only the first isolate. I would say the same should have been done for the prevalence of carriage (since the sensitivity for finding a positive results is higher when taking more parallel observations per individual). Or at least discuss the impact of a different number of isolates between individuals. – **We feel that we have sufficiently discussed the potential impact of different isolates per individual and the ways in which we addressed possible bias due to the variation in outcome measurements. Please also see comments below.**

Results
This part has changed massively. However, I still am confused sometimes about the numbers. In line 332-334 is stated how many isolates were collected from how many children from how many households. By including the number of isolates where cephalosporin resistant it get's less clear. I would not put in outcome results mixed with output results. – **We believe that specifying how many cephalosporin-resistant isolates were cultured from fecal samples is key information for the reader that should be presented early in the results section.**
Line 377 and further: In how many of the children with at least one positive isolates, positive isolates were also found in animals? –

Line 388: how were the 200 additional isolates selected? – **We have clarified that these additional 200 isolates were randomly selected.**
Line 406: please delete 'all fecal samples', because you are modeling at the isolate level. – **The denominator here includes all fecal samples, including those without a 3GCR-EC isolate, which were characterized as not having the outcome of interest. Therefore we will leave in “all fecal samples”.**
Line 419: do you mean marginally significant? Instead of marginally significant I would suggest to use the term borderline significant. – **“Marginally associated” and “borderline significance” are both commonly used. We are happy to change to borderline regardless.**

If I understood correctly, another manuscript is discussing the similarity between the isolates collected within epidemiologically linked clusters (humans and animals from the same household). The combination of an epi analysis and the isolate characteristics are telling a much fuller view on the matter. I really don't understand why this information is not used in this manuscript to support the findings. – **This previously published manuscript used only a subset of the full study dataset presented in the current manuscript under review. We believe that citing the previous manuscript in the discussion section of the current paper succinctly links the two analyses and their findings. The current manuscript aimed to focus on an epidemiological risk factor analysis and the included analyses are already quite substantial, so we respectfully disagree that these previously published findings from a different analysis and subset of data should be included in the current manuscript.**

Discussion
Line 445-449: Please delete, you don't have to summarize the study. – **We have deleted the restated methods at the beginning of the discussion section.**
Line 461: Depending on the ARB/ARG, but most of the studies do not report transmission from humans with intense contact with animals to other humans. You might want to nuance this statement a bit. **– We have updated this sentence to say “associations between” rather than “link” to clarify that statistical associations are the key method, not characterization of transmission.**
469-474: Please delete, I don't see any reason to state this. It does not interpret results or highlight any finding in a broader context. – **These sentences clarify how our study improves upon previous epidemiological studies that have attempted to address similar research questions. We believe this is important for placing our research in context with the literature and highlighting the contributions of this work to the field.**
Line 543: I don't agree with your reasoning regarding your choice on what level to perform the analysis. Especially in terms of translating and interpreting the results. A risk factor for a positive isolate is less interesting, since the exposure is present (and therefore also focus for mitigation) on the individual level. And not on the isolate level. I would strongly suggest (as the other reviewers did as well) to model at the individual level and do a sensitivity level as you did with an individual outcome only based on the first isolate. Furthermore, I find the term 'some slight differences' rather vague. – **Given that other reviewers and editors have no further concerns about the approach used after reviewing the previous responses and given the inclusion of the sensitivity analyses, we are confident that our analytic methods & approach are appropriate. While the findings may be ‘more interesting’ or interpretable at the individual level, our concerns around bias due to variation in outcome measure frequency remain. As previously mentioned, other epidemiological studies using isolate-level data have used a similar isolate-level approach.**

Line 546: Please replace 1 by one. – **This has been replaced.**
Line 566-594: Although I understand the request for including implications, I find this way too long. I am sure you can describe the points you are raising in a much more concise way. – **We believe each sentence in this paragraph provides a specific and distinct recommendation for policy & the implications of such policies. We believe this section is clear and concise given the breadth of potential policy implications of this work.**


Reviewer #4: The authors have addressed most of my points but there remain a few to clear up.

I asked whether there had been a formal sample size determination but the rebuttal does not seem to mention this either way. – **Apologies for omitting this in the previous response to comments – we have added a thorough explanation of power calculations and sample size for the parent study in the Supplemental Materials. We have also added the following sentence in the Methods under Study Design: “For power calculations and sample size determination, please see Supplemental Materials (S2. Sample Size & Power Calculations).”**

The authors have estimated parameters in their model page 18 "Multivariable log-binomial regression models were used to estimate unadjusted and adjusted
relative risks" so I still fail to see how they can continue to claim they used non-parametric methods and hence how these were supposed to deal with selection effects. **– As described in the statistical analysis section in the methods, we used Generalized Estimating Equations, a semiparametric estimation method which produces less biased inference compared to other modeling techniques that rely on assumptions of the data-generating distribution and model specification. (Hubbard et al., 2010, Epidemiology) However, this approach is only semiparametric because we still assume an underlying data distribution, so the text has been updated to reflect that our approach is semiparametric, not nonparametric.**

I am afraid I do not understand their response about Supplementary Figure S1. Either a risk factor is theoretically important in which case it belongs in S1 and in the analysis even if it fails to reach some arbitrary level of statistical significance or it is not in which case it should not appear anywhere. The authors have put household members use of antibiotics in S1 but not the analysis which is inconsistent. –**Apologies for the confusion on this figure. The figure should only include “recent child antimicrobial use”, as this is the risk factor of primary concern and is most likely to impact a child’s current ARB carriage status. The figure has therefore been changed accordingly – thank you for pointing this out!**
