# Supplementary material for: Risk factors for extended-spectrum beta-lactamase (ESBL)-producing E. coli carriage among children in a food animal-producing region of Ecuador: A repeated measures observational study
Source: PLoS Med. 2023 Oct 13;20(10):e1004299. doi: 10.1371/journal.pmed.1004299 (PMC10621961; doi:10.1371/journal.pmed.1004299)
Supplement: Attachment — Submitted filename: PLOS Medicine Reviewer Comments.docx [file pmed.1004299.s003.docx]

PLOS Medicine Reviewer Comments

-----------------------------------------------------------
Requests from the editors:

GENERAL
1. Please respond to all editor and reviewer comments below, in full.
2. Please number each line of the manuscript starting with 1 on the first line of page 2 and in continuous sequence thereafter. – **Line numbers have been added beginning on page 2.**
3. Please ensure that the study is reported according to the STROBE guideline, and include the completed STROBE checklist as Supporting Information.  Please add the following statement, or similar, to the Methods: "This study is reported as per the Strengthening the Reporting of Observational Studies in Epidemiology (STROBE) guideline (S1 Checklist)." The STROBE guideline can be found here: <http://www.equator-network.org/reporting-guidelines/strobe/> - **This statement has been added to the Methods section (paragraph 1) and the STROBE checklist has been added to the supplemental materials.**
**4. When completing the checklist, please use section and paragraph numbers, rather than page numbers.**

ABSTRACT
Abstract methods and findings:
1. Please include the actual amounts and/or absolute risk(s) of relevant outcomes (including NNT or NNH where appropriate), not just relative risks or correlation coefficients. (example for absolute risks: PMID: 28399126). -

2. Where 95% CIs are reported please also report p-values.  – **We have added p-values where necessary.**

3. Suggest for clarity that statistical information is defined clearly perhaps with abbreviations (e.g. 95% CI) at each reporting, as you have done in the main manuscript, as opposed to just once in full. – **Abbreviations for statistical results have been clarified and added at each reporting.**

4. Please ensure that all numbers presented in the abstract are present and identical to numbers presented in the main manuscript text. – **We have double checked that all numbers are consistent with the main text.**

5. It may be helpful to elaborate on the details of the study design including for clarity some of the different risk factors (or categories of risk factor) investigated.  The reader doesn’t learn of any until the significant results are reported.  Suggest including the number of participants (as well as the sample number), length of follow-up/number of visits and more clearly defining the region (see also statistical reviewer comments) and use of animals for subsistence farming Vs trade – **Thank you for these suggestions. The abstract has been revised accordingly to include categories of all risk factors investigated (see Background), the number of children and samples, the number of visits, and length of follow-up. Unfortunately we do not have data on whether domestic livestock were used for subsistence farming vs. trade.**

6. Suggest moving the following sentence “We used multivariable log-binomial regression models to estimate relative risks (RR) of 3GCR-EC and ESBL-EC carriage.” To follow the description of the study before the results are presented. – **This sentence has been moved accordingly, thank you for the suggestion.**

7. Please include the important dependent variables that are adjusted for in the analyses. **–** **We have revised the following sentence to include covariates included in models: “We used multivariable log-binomial regression models…adjusting for child sex and age, caregiver education, household wealth, and recent child antibiotic use.”**

8. In the last sentence of the Abstract Methods and Findings section, please describe the main limitation(s) of the study's methodology - **We have added the following sentence at the end of the Abstract Methods and Findings section: “The primary limitations of this study are the use of proxy and self-reported exposure measures; future studies should characterize transmission pathways by assessing antibiotic resistance in commercial food animals and environmental reservoirs.”

Abstract Conclusions:**
9. Please address the study implications without overreaching what can be concluded from the data; the phrase "In this study, we observed ..." may be useful.

10. Please interpret the study based on the results presented in the abstract, emphasizing what is new without overstating your conclusions.

11. Please avoid vague statements such as "these results have major implications for policy/clinical care".  Mention only specific implications substantiated by the results.
12. Please avoid assertions of primacy ("We report for the first time....")

**We have revised the conclusion based on the above statements as follows: “In this study, we observed an increase in enteric colonization of antibiotic resistant bacteria among children with exposures to domestic animals and their waste in the household environment, and children living in areas with a higher density of commercial food-animal production operations.”**

**AUTHOR SUMMARY**
1. At this stage, we ask that you include a short, non-technical Author Summary of your research to make findings accessible to a wide audience that includes both scientists and non-scientists. The Author Summary should immediately follow the Abstract in your revised manuscript. This text is subject to editorial change and should be distinct from the scientific abstract. Please see our author guidelines for more information: <https://journals.plos.org/plosmedicine/s/revising-your-manuscript#loc-author-summary>

**METHODS and RESULTS**
1. Did your study have a prospective protocol or analysis plan? Please state this (either way) early in the Methods section.
a) If a prospective analysis plan (from your funding proposal, IRB or other ethics committee submission, study protocol, or other planning document written before analyzing the data) was used in designing the study, please include the relevant prospectively written document with your revised manuscript as a Supporting Information file to be published alongside your study, and cite it in the Methods section. A legend for this file should be included at the end of your manuscript.
b) If no such document exists, please make sure that the Methods section transparently describes when analyses were planned, and when/why any data-driven changes to analyses took place. 
c) In either case, changes in the analysis-- including those made in response to peer review comments-- should be identified as such in the Methods section of the paper, with rationale.

**We have added the following statement to the Methods section (paragraph 1) regarding our analysis plan: “Analysis plans for this manuscript were developed beginning in May 2020 and were last revised in March 2021 after sequencing results became available; we amended our analysis plan to include descriptive results of antibiotic resistance genes identified from sequenced E. coli isolates.”**

2. For all observational studies we ask that the following is clearly indicated in the manuscript text:
(1) the specific hypotheses you intended to test,
(2) the analytical methods by which you planned to test them,
(3) the analyses you actually performed, and
(4) when reported analyses differ from those that were planned, transparent explanations for differences that affect the reliability of the study's results. If a reported analysis was performed based on an interesting but unanticipated pattern in the data, please be clear that the analysis was data-driven. - **We have double checked that these are clearly indicated in the main text.**

3. Page 11 para 3: please ensure that where you report percentages that the numerators and denominators used to derive them are clearly reported.  In the paragraphs above whole numbers are reported but it is a bit arduous to the reader to go back and forth for reference. **– Thank you for this suggestion. We have clarified the denominator of a percentage reported in paragraph 2 of the Methods section as follows: “Two percent (40/1,739) of child fecal samples…”. We have also added in the beginning of paragraph 3 the total number of households included in the study (“In a majority of the 594 included households…). Because Table 2 includes the raw data for characteristics reported in this paragraph, we didn’t want to add the numbers and denominators for each characteristic in the text to improve readability. We would prefer to keep as is, but can add numbers and denominators if the editors feel that is best.**

FIGURES
Please consider avoiding the use of red or green to make the figures more accessible to those with colour blindness
Figure 1: Please confirm that the appropriate usage rights apply to the use of this map. Please see our guidelines for map images:
<https://journals.plos.org/plosmedicine/s/figures#loc-maps> – **Yes, we created this map and have usage rights. We have updated the Figure caption with the appropriate attribution statement.**

Figure 2: PLOS medicine usually advises against the use of pie charts but in this specific case we agree with the methodological reviewer that they serve their purpose here very nicely – **Thank you!**

Figures 3 and 4: Please clearly indicate the meaning of lines and dots on the plots.  Please clearly indicate whether the analyses are adjusted and in the figure captions what factors they are adjusted for. Where adjusted analyses are presented, please also present unadjusted analyses. It would be helpful to the reader to list the numerical values for RR and 95% CIs in a column next to the plots.  In addition, where you report 95% CIs please also report p-values and in the figure caption please define the test used to derive them – **Figure 3 has been replaced since we believe it did not significantly add to results already reported in tables. Figure 3 is now a barplot of the joint distribution of exposure and outcome variables, based on suggestions from reviewers.** **We have updated figure 4 accordingly and added p-values in supplemental tables with the effect estimates and confidence intervals for these figures.**

Please ensure all abbreviations for statistical reporting are defined e.g RR and CI – **We have double checked our abbreviations for statistical reporting and defined them where necessary in figures and tables, and specified that analyses were adjusted when appropriate. Because our analysis is already a bit complex, we have made unadjusted results available in supplemental materials.**

TABLES
For the main outcome measures presented in table 3 and 4, to help facilitate transparency of data reporting please indicate in the column headers that these analyses are adjusted and also provide the unadjusted analyses for comparison. – **Thanks for catching this; we have added ‘adjusted RR’ to column headers. We have added unadjusted results, as well.**

SUPPLEMENTARY FIGURES AND TABLES
Please check carefully throughout and ensure all abbreviations are defined (for example SES, ESBL). – **We have double checked that all abbreviations are now defined.**

Where you report 95% CIs please also ensure p-values are reported – **P values have been added where appropriate.**

Where adjusted analyses are reported please clearly indicate which factors are adjusted for, in the caption, and also present the unadjusted analyses for comparison. – **Details on adjusted analysis have been clearly indicated in supplemental figures and tables.**

DISCUSSION
Please present and organize the Discussion as follows: a short, clear summary of the article's findings; what the study adds to existing research and where and why the results may differ from previous research; strengths and limitations of the study; implications and next steps for research, clinical practice, and/or public policy; one-paragraph conclusion. – **We have double checked that our discussion is organized as described here.**

REFERENCES
Please use the "Vancouver" style for reference formatting, and see our website for other reference guidelines
<https://journals.plos.org/plosmedicine/s/submission-guidelines#loc-references>

Citations should be in square brackets, and preceding punctuation [1,3,5,7] or [1-4,7]. Please note the absence of spaces between citations.

Journal name abbreviations should be those found in the National Center for Biotechnology Information (NCBI) databases.

In the bibliography, please list up to but no more than 6 names followed by et al where more than 6 authors contribute to a study.

**References and citation formats have been updated accordingly.**

**PARACHUTE RESEARCH**
We note that you conducted research or obtained samples in a foreign country. Did you consider including a local author as first or last author? If not, we recommend that you consider doing so in line with ICMJE's authorship requirements (<https://www.icmje.org/recommendations/browse/roles-and-responsibilities/defining-the-role-of-authors-and-contributors.html>).   PLOS has a parachute research policy which aims to promote collaboration and inclusivity in global health research. You are required to complete PLOS’ questionnaire on inclusivity in global research and submit it with your revised paper. The policy and questionnaire can be found at <https://journals.plos.org/plosone/s/best-practices-in-research-reporting>. – **Thank you for this important suggestion. We wholeheartedly agree that local researchers should share leadership roles as first and last authors. Prior to beginning this research and throughout the study, we established clear roles and expectations with our local partners at USFQ about who would lead which analyses and lead manuscript writing. Our colleagues at USFQ have published as first authors on other manuscripts that pertain more to their skillsets (i.e. microbiology and molecular biology), with the lead investigator at USFQ, Gabriel Trueba, as final author (e.g., Salinas et al., 2021, EHP). This manuscript uses an epidemiological approach and was therefore led by a UC Berkeley researcher with expertise in epidemiology (Amato), with support from her doctoral advisor (Graham), who served as final author for this particular manuscript.**

DECLARATION STATEMENTS
Please remove these from the end of the manuscript and place in the manuscript submission form only.  The ethics statement can be placed in the methods section. – **These statements have been removed from the end of the manuscript. The statement on funding was instead added to the end of the first paragraph of the Methods section. The statement on sequencing data availability was added to the Methods section, subsection DNA Sequencing and Analysis.**

SOCIAL MEDIA
In the event that your paper is published, to help us extend the reach of your research, please provide any Twitter handle(s) that would be appropriate to tag, including your own, your coauthors’, your institution, funder, or lab. Please respond to this email with any handles you wish to be included when we tweet this paper. - **@HeatherKAmato, @liss_salinast, @IMUSFQ, @USFQ_Ecuador, @UCB_EHS, @UCBerkeleySPH**

--

Comments from the reviewers:

**Reviewer #1:**

This manuscript describes the results of an observational study on environmental exposure by commercial and small-scale food animal production and risk factors for ESBL-producing E. coli and third-generation cephalosporin-resistant E. coli carriage in children in Ecuador. It is important that this kind of studies are performed in low- and middle-income countries to increase the knowledge and to identify potential measures that can be taken to reduce the risks for ESBL carriage in children. Furthermore, it is a nicely designed and well-written study with repeated measurements per household. I do have some questions for the authors though.

Major comments:
1.      Why were the selected E. coli isolates frozen and thawed again before susceptibility and phenotypic ESBL-production testing was performed? Do the authors think this may have influenced the results they found compared to immediate susceptibility and phenotypic ESBL-production testing? – **The isolates were freeze-thawed due to the preliminary screening step of growing ceftriaxone-resistant *E. coli.* We waited to determine which isolates were ceftriaxone-resistant, then only performed susceptibility/ESBL phenotypic testing on samples that grew on media with ceftriaxone. To clarify, we have added in the Methods under subsection Microbiological Methods, “Up to five ceftriaxone-resistant isolates phenotypically matching *E. coli* were selected from each fecal sample and preserved…”. (line 236) We do not believe that a single freeze-thaw cycle would influence the results of phenotypic antibiotic resistance and ESBL production.**

2.      Why were the analyses performed on isolate level, and not on child-level (child being colonized or not)? Did the authors check for results on child-level? I am also curious on parish level how many kids were colonized, and the mean/sd number of isolates on child-level per parish. – **To your first point, we considered whether to analyze at the person-level or isolate level and came to the conclusion that analyzing multiple isolates will give a) us more accurate outcome information about each individual, and b) will be less biased due to the imbalanced nature of the number of isolates assessed per person per time point. As mentioned in the methods section, each individual may have had up to 5 3GCR-EC isolated from their stool sample, but the number of isolates varied from 0-5. For an individual who had 5 isolates, the probability of detecting ESBL is greater than it would be for an individual who only had 1 isolate. Our repeated measures analysis accounts for the fact that isolates across each time point are grouped by the individual (i.e., the analysis is clustered at the individual level). Existing studies assessing antibiotic resistance of multiple bacterial isolates per sample/person have also conducted regression analyses at the isolate level (e.g., Caudell et al. 2018, The Lancet Planetary Health), and we believe this is the right approach for our analysis.**

3.      How were the isolates selected that were sequenced? It is not mentioned in the Methods section how this selection was done. – **We have added the following sentence for clarification: “We analyzed whole-genome sequencing data for a subset of 571 3GCR-EC isolated from fecal samples of the 365 children that were 3GCR-EC carriers. We first selected one isolate per child for sequencing, then aimed to select an additional 200 isolates to include more ESBL-EC in sequencing analyses.” (lines 391-394)**

4.      Page 11 second paragraph: 1,677 child fecal samples from 594 households remained. Is it then correct that the 1,677 fecal samples were taken from 594 children? Also, I don't get how the numbers mentioned in this paragraph add up to 1,940 observations: is that 904 3GCR-EC isolated in children plus 1,060 3GCR-EC in animals (=1,964)? Please clarify. – **Thanks for letting us know this wasn’t clear! To your first point, yes, the 594 households = 594 children. We updated this paragraph specifying these are “children from different households”. With a varying number of multiple isolates per fecal sample, we ended up with 1,940 observations across the 1,677 fecal samples. The results section on page 11 has been revised for clarification: “After removing children with missing exposure, outcome, and covariate data from household surveys, 904 3GCR-EC isolates from 1,677 child fecal samples from 594 children remained. This resulted in a total of 1,940 observations (including multiple isolates per fecal sample) in the final dataset for the primary statistical analysis.” (line 372)**

5.      A lot of different statistical analyses and tests were performed. However, I did not read anything about corrections made for multiple testing which I think is very appropriate in this case. – **This is a great point. However, we are confident that our estimates and standard errors are sufficiently robust since we used GEE with exchangeable working correlations to produce very conservative confidence intervals.**

6.      Table 1: Is it correct that none of the household food animals received antibiotics in the 5th data collection cycle? In the other cycles it ranged from 9.5-26.4%, so this is a big difference. Do the authors have an explanation for this finding? – **Yes, this was the case based on caregiver reporting. We also noticed that this is a very big difference and wanted to investigate further, in a separate analysis. We believe that antibiotic use changed significantly due to many factors related to the COVID-19 pandemic. We have a separate manuscript (under review elsewhere) that addresses these longitudinal changes in antibiotic use and carriage of resistant bacteria as they relate to infection control policies and public health messaging established in Ecuador after the onset of the pandemic.**

7.      Longitudinal findings in individual children, did the authors analyse whether the same children carried the same ST E. coli and gene during follow-up data collection cycles? Of course, this is only possible for children who participated >1 cycle. This would also be very interesting to see, whether there was a new or new type of colonization or whether it is still the same type. In the last case, it is could be a non-cleared colonization or a repeated exposure/colonization. – **Thank you for this suggestion! This was a consideration for this analysis, but we decided it was beyond the scope of the current manuscript. One of our colleagues at USFQ is working on assessing longitudinal colonization of specific *E. coli* STs within individuals for a separate paper.**

8.      Table S6: high percentage of OXA genes in Tababela compared to all other parishes, explanation for this difference. – **It’s difficult to make any inferences about the cause for OXA genes being detected more frequently in Tababela, especially given the small number of isolates in this parish (N=16).**

9.      Figure 2/Table S8: most of the commonly detected CTX-M-type genes are both found in parishes with low- and high-CFOs. For example, CTX-M-15 represents 50% of the CTX-M genes found in Tababela with low CFO and ≈55% in El Quinche with high CFO. Are there any other suspected common sources that the authors could think of which may explain these findings? – **We think the irrigation canals are potentially transporting commercial food animal waste throughout the study site due to runoff. The irrigation canals span parishes with and without CFOs, and we have identified high levels of fecal contamination in irrigation canals throughout these parishes as well. We have added the following sentences to the discussion section: “We also reported increasing relative risks of ESBL-EC for children in households that both owned food animals and were close to a commercial food animal operation drainage path, which often feed into irrigation canals. Furthermore, the irrigation canals run throughout the study site, which may, in part, drive the geographic spread of beta-lactamase genes we detected in parishes with both high and low numbers of commercial production facilities. ” (lines 741-746).**

10.     Results page 14/15 and 1st paragraph of the Discussion. All results with an RR >1 are presented as having an increased risk, but some of them are not statistically significant since the 1 is included in the 95% CI. In my opinion this should be mentioned more explicitly when results are or are not statistically significant. For example: "However, the combination of owning household food animals and living <1.5 km from the nearest commercial operation was associated with an increased risk in 3GCR-EC carriage (RR: 1.15; 95% CI: 0.97, 1.33) compared to those without food animals who lived further from commercial food animal operations". It is presented like a significant increased risk, but it is not. Also, the last line of page 14: "chicken ownership (RR: 1.10; 95% CI: 0.98, 1.24), and rarely/never washing hands after contact with animals (RR: 1.15; 95% CI: 0.98, 1.34)". These are non-significant risk factors while the first two factors mentioned in this sentence are not. I would not mention them separately as these non-significant factors can be found in the Tables. Finally, this non-significant "rarely/never washing hands after contact with animals" is even mentioned in the abstract together with significant results. – **Thank you for articulating these concerns. We have removed descriptions of non-significant results from the abstract and results, and have specified ‘marginally’ significant where necessary. We believe this helps address other comments about presenting results more concisely, as well.**

11.     Table 4: The RR of in children without household food animals with distance to the nearest farm <1.5km away seems to be protective (although not statistically significant) compared to >=1.5km away. Any explanation for that? – **Yes, we agree this is unexpected! However, the confidence intervals for these estimates are quite wide so it’s difficult to make any inferences here.**

12.     Page 17 line 2: Were animal samples and child samples matched? So were the same ST's and genes identified in children and animals of the same household? And were analyses performed on these matched isolates? – **We have a separate manuscript on clonal *E. coli* sharing between animals and children of the same household, and between different households - see Salinas et al., 2021 EHP.**

Minor comments:
1.      Page 3 last paragraph: "ESBL-producing enterobacteriaceae infections". "Enterobacteriaceae" are renamed as "Enterobacterales". – **Thanks for catching this, ‘enterobacteriaceae’ has been changed to ‘enterobacterales’ throughout.**

2.      Table S3: The column name of the second column is 'H', I think this reflects the number of households. The legend says it should read 'HH'. Please adapt them consistent. – **Thanks for this, H has been changed to HH in the table column name.**

3.      Table S10: I suggest to present the RR and 95% CI with 2 decimals (like in all other Tables) and the P-values with 3 decimals instead of 4. – **Table S10 has been updated accordingly.**

--

**Reviewer #2:**

Dear editor,

Thank you for asking me to review this article. I believe the topic fits Plos Medicine really well. Livestock is already known to be a reservoir for antimicrobial resistant bacteria and livestock-human transmission is documented as well. However, most of the studies are conducted in the Western world. I believe it is very interesting to investigate the risk of animal food production for ESBL carriage in humans in other parts of the world. Therefore, I do believe that after improvement this manuscript can add valuable information to the topic of human antimicrobial carriage. However, I have some major concerns regarding the data analysis performed, interpretation of the results and following conclusions stated. Therefore, I would advise major revisions.
I would recommend that the authors check the data analysis and thoroughly read through their manuscript again and rewrite parts of it and also reduce the length of the manuscript. In the section "Comments to the authors" I also commented in more detail regarding abovementioned aspects.

Comments to the authors

I agree with the authors that it is very important to investigate livestock as a risk factor for human carriage of antimicrobial resistant bacteria (or genes) in low and middle income countries, therefore I stress the importance of the topic. However, I have some concerns regarding certain aspects (mainly your data analysis and discussion) of the manuscript. Moreover, I believe you can shorten the manuscript. Below you will find some comments in more detail.

Abstract
Page 2, Conclusions: although I understand the reasoning of what is stated in these conclusions, I don't think this conclusion aligns well with the study conducted. Risk factors for carriage were under investigation, not policies and interventions to curb the spread. – **Thank you for this comment, we have updated the conclusion section to better represent the specific findings in our study: “In this study, we observed an increase in enteric colonization of antibiotic resistant bacteria among children with exposures to domestic animals and their waste in the household environment, and children living in areas with a higher density of commercial food-animal production operations.”**

Introduction
1. I find the introduction too long. Every paragraph should be shortened. I think the order of paragraphs can be improved – **We have cut sentences, combined sentences, and removed words/phrases to be more succinct to shorten the intro.**

2. First paragraph, first sentence: I don't understand what you state. Contamination with what? You might consider deleting this first sentence. – **Thank you for letting us know this sentence wasn’t clear. We have clarified that we mean ‘environmental fecal contamination’ from food animal production.**

3. First paragraph, ref Seifert et al. 2013: There is more recent work done that you can cite. I would also use more references for such a statement. – **We have added a recent review (Ma et al. 2021) to further support this statement.**

4. First paragraph, last sentence: Please specify which communities. Moreover, although livestock-human transmission is important, but I would mention human-human transmission as background information as well. – **Thank you for these suggestions. We have specified “LMIC communities” in the last sentence (lines 66-67). With respect, we do not think discussing human-human transmission is necessary in the introduction given the focus of this study, and given that the introduction should be shortened.**

5. Second paragraph: this part described the urgency and importance. I would place this paragraph first. This is the reason why you want to investigate risk factors for this human carriage. – **We respectfully disagree and would like to keep the order of these paragraphs as is. The first paragraph addresses the overarching global health issue at hand: the spread of antibiotic resistance associated with food animal production**

6. Third and fourth paragraph can be combined.- **Thank you for this suggestion, we have combined these paragraphs and shortened the paragraph length with minor edits.**

Methods
7. The first paragraph could use the subheading 'study design'. I think the section on data analyses is hard to follow. I would suggest the authors to be more clear and precise. A figure might help to visualize it. – **Good suggestion, the subheading ‘study design’ has been added, as well as a brief section describing the study site. We’ve also reorganized the statistical analysis section to improve clarity.**

8. Page 5: What is the reasoning for the inclusion criteria 'child between 6 months and 5 years'? – **Typically children <6 months are breastfeeding, are not very mobile, and don’t interact with their environment (including animals) in the same way that older children do. These factors impact their nutritional status, gut microbiome, risk of infection, and make them systematically different than children >6 months. Including children <5 years (excluding young infants) is common practice for studies of enteric infections due to the increased risk of infection and associated adverse health outcomes among this age group.**

9. Page 6, 'Density…..(Amato et al. 2020)': This is background information/interpretation and should not be discussed in method section, but in introduction and/or discussion. – **We respectfully disagree with this suggestion. Decisions about the analytic approach are commonly justified with existing literature in the methods sections; we believe this background information is immediately relevant to the reader in order to support our methodological choice of using density as a measure of exposure.**

10. Page 7, outcome assessment: What do you mean by animal that were present at the household? Only domestic animals? Or food production animals (backyard farming) as well? – **This has been clarified and now reads “domestic or food animals”.**

11. Page 9, statistical analyses: How did you account for potential interaction? What do you mean by three different models? – **We have significantly revised the statistical analysis section to improve clarity on the types of models used and how interaction was accounted for (we added an interaction term in models).**

12. What do you mean by 'using cut-points ….. policy relevance'? – **We revised this to say ‘interpretability’ rather than policy relevance.**

13. How where the results from the animal derived isolates used in the models? – **Whether or not animal stool was positive for 3GCR-EC/ESBL-EC was analyzed as a secondary risk factor.**

14. What do you mean by assessed at the isolate level? Did you model at the isolate level? I would say it makes sense to model at the child level (= sample level). – **Yes, we modeled at the isolate level but adjusted for clustering at the individual level.** **We considered whether to analyze at the person-level (i.e., aggregate samples per child and time point to yield a single outcome measure, presence/absence of any 3GCR-EC/ESBL-EC) or isolate level and came to the conclusion that including multiple isolates will give a) us more information about each individual’s colonization status, and b) will be less biased due to the imbalanced nature of the number of isolates assessed per person per time point. Our repeated measures analysis accounts for the fact that isolates across each time point are grouped by the individual (i.e., the analysis is clustered at the individual level, not the isolate level). Existing studies assessing antibiotic resistance of multiple bacterial isolates per sample/person have also conducted regression analyses at the isolate level, and we believe this is the right approach for our analysis.**

Results
1. I think the results section can be shorter in general. Lot of percentage and gene types are described, which can easily be seen in the Table. I would focus on highlights in text. – **Thank you for this suggestion – the results section has been revised to be more concise throughout.**

2. Page 10/11: I find the figure S2 helpful in understanding. However, from both the text and the figure I cannot interpret how many households did how many repeats? Please provide this information? – **Great point, we have added the following sentence in the first paragraph of the Results section: “The median number of household visits (i.e., fecal samples) for each child was three; 23% of these 600 households were visited five times, 18% four times, 11% three times, 11% two times, and 37% were visited once.”**

3. Page 11: What are the 1940 observations? It looks like you are modeling at the isolate level. But exposures are at the child sampling moment level (i.e. each stool sample is a single observation). – **You are correct that exposures are at the child/time point level, and the outcome is at the isolate level. With a varying number of multiple isolates per fecal sample, we ended up with 1,940 observations across the 1,677 fecal samples. The results section on page 11 has been revised for clarification: “After removing children with missing exposure, outcome, and covariate data from household surveys, 904 3GCR-EC isolates from 1,677 child fecal samples from 594 children remained. This resulted in a total of 1,940 observations (including multiple isolates per fecal sample) in the final dataset for the primary statistical analysis.” (line 372)**

4. Page 12, Food Animal Production & Domestic Animals, first paragraph: what is the rationale for only mentioning the first four cycles of data collection to describe the distance to the number of farms? – **We now also mention the proportion of households within 5km of 6 or more commercial food-animal operations in the fifth cycle: “…this dropped to 43% in the fifth cycle”. (line 422)**

5. Page 14, Risk Factors for 3GCR-EC: I am slightly confused by these results. You state that children with > 5 commercial food animal operations in a 5-km radius of their household had a higher risk of 3GCR-EC carriage. However, in table 3 I only see the results of the stratified analyses. Without stratifying, what is the effect? In order to understand any effect modification, I would like to see the overall results as well. Why is a different cut-of chosen for the no of operation in an 5 km radius in the analysis among those with household food animals. Also, I am missing the sizes of the strata. – **We have added main effects results to improve interpretation of results, both with and without consideration for interactions – please see Table 3. The interaction tables are not only stratified results (i.e., “within strata”). For distributions of each outcome within strata, please see the newly added Figure 3.**

Discussion
1. I think most of the paragraphs in the discussion can be shortened, it often takes a long read before you reach your message. The paragraph on policy advice should be deleted in my opinion. This was not your research question in the first place, and I think your results should be used for policy if applicable, but policy is not supposed to be made by researchers. – **Thanks for this feedback. However, we respectfully disagree and believe that some discussion of the policy relevance and potential implications of this research is merited. In fact, the editors request that we discuss “implications and next steps for research, clinical practice, and/or public policy” in the discussion section.**

2. Page 16, second paragraph: I do not think that the epidemiological studies have shown a clear link between food animal production and community-acquired antibiotic-resistant infections. Instead it is associated with carriage of ABR bacteria. – **This sentence has been updated: “Epidemiologic studies have established a clear link between commercial food animal production and ARB carriage…”. (lines 637-638)**

3. Page 17, top of page: I agree that repeated measurements are essential to investigate this association, but from the manuscript the range of number of repeated measurements is not clear. – **We have added the following sentence in the first paragraph of the results section: “The median number of household visits (i.e., fecal samples) for each child was three; 23% of the 600 households were visited five times, 18% four times, 11% three times, 11% two times, and 37% were visited once.” (lines 367-369)**

4. Also, you mention your One Health design, what do you mean by that? I read that you samples animals (domestic or commercial as well?) at the same household, but I don't see how you used it in your analysis. – **We feel that this work is a clear example of a One Health study given our focus on the human health impacts of exposures to animals at the household and community level, both through direct contact and through indirect environmental pathways (e.g. contact with irrigation water that is contaminated with animal waste). The animal samples were used to identify 3GCR/ESBL-E. coli in animal feces found at individual households, which was then used as a potential risk factor for child carriage of 3GCR-EC/ESBL-EC (see Figure 4).**

5. Page 17, paragraph on AB use: I find this paragraph hard to follow. Please be clear on what is from the other study and what is from your study. – **We have clarified the distinction by specifying that the other studies are ‘other analyses of data collected for this cohort study’. (line 694)**

What do you mean by indirect curbing of infectious diseases in animals by the pandemic? – **The COVID-19 pandemic led to infection control policies that reduced transmission of other bacteria and viruses in humans. We know that strain-sharing is between humans and animals is common, as described in this paragraph, and when human transmission decreases we would expect to see animal transmission decrease. To avoid confusion we removed this statement about indirectly reducing transmission in animals.**

Page 18, bottom of the page: I don't agree with your analysis at the isolate level (see comments on methods and results) – **Please see above comments regarding this point.**

Page 19, paragraph on prevalence. I would move this paragraph up, since it is a rather important finding. – **Thanks for this suggestion, this paragraph has been moved up (third paragraph in discussion).**

Page 20, gene types found in children: I understand that the gene types found are similar as in livestock animals. What are the main types in the general population lesser in contact with animals? That should give a more or less control situation. – **There is no simple answer to this! There are many beta-lactamase genes prevalent in different parts of the world, and many that are specific to South America. We’re hesitant to compare our results to other studies in Ecuador or South America that took place in different regions or collected samples from different sources (i.e. hospital-based data) because we wouldn’t be able to say for sure what the level of animal contact is in those other studies. Small-scale animal production and contact with domestic animals is common throughout Ecuador and South America.**

Page 20: delete the paragraph on policy and please remove the policy regarding statements also from your conclusions and abstract. – **Thanks for this feedback. However, we respectfully disagree and believe that some discussion of the policy relevance and potential implications of this research is merited. In fact, the editors request that we discuss “implications and next steps for research, clinical practice, and/or public policy” in the discussion section. We have removed policy statements from the abstract, however.**

**--**

**Reviewer #3:** This is a potentially interesting study on a relevant topic. However, it is presented as an epidemiological study, which highlights a number of shortcomings in the design and reporting in the paper.  The study mostly uses proxy indicators of AMR exposure, or caregiver reported indicators of hygiene and child behaviour. Any observed associations do not really shed light on the pathways of AMR transmission.
The key limitation is the lack of assessment of antibiotic resistant E. coli in the majority of  the exposures that are reported.


Specific comments:
Assessment of risk factors is done using proxy measures (e.g. geographical distance and concentration of commercial livestock/animal farms) without any direct measures of antibiotic resistant bacteria, genes or antibiotic residues and there is no information presented on whether these commercial premises used antibiotics, which antibiotics were used, and/or gut colonisation with ARB in the commercial animals. – **These are excellent suggestions and we agree! However, collecting fecal samples at commercial production facilities or other environmental samples to characterize transmission pathways was not in the scope of this study. We have updated the discussion section on page 19 as follows: “there is a lack of available data on antibiotic usage and resistance in intensive, commercial food animal operations and their effluent in Ecuador due to limited oversight and surveillance. Unfortunately collecting this data was not within the scope of this study. To accurately characterize the extent to which antibiotic use in commercial production drives community-level transmission antibiotic-resistance, future studies should investigate antibiotic use and antibiotic-resistant bacteria in food animals, food animal production waste, nearby environmental reservoirs, and food animal products.” (lines 794-802)**

Similarly, the proxy measure of proximity to drainage channels does not provide direct evidence of exposure to ARB/ARG. No analysis of water samples from these drainage channels were taken or analysed. – **Another great point, and something our research team piloted in this study site (only identified fecal indicator bacteria). Initial pilot data indicated that low-lying communities have very high levels of fecal contamination, while higher elevation communities have low/undetectable levels of fecal contamination using quantitative fecal indicator bacteria tests. I have added a sentence on irrigation canal contamination in the discussion section: “For example, animals may be exposed to high levels of fecal contamination in irrigation canals , though household animals drinking irrigation water was not identified as a significant risk factor for human ARB carriage in our study” (lines 735-738).**

**Our team recently received a larger grant to collect and analyze surface water samples from irrigation canals and streams throughout the study site. We will characterize antibiotic resistant bacteria in these waterways as part of this new study.**

Proxy measures can only provide weak inferences about relationships (or not) between environmental exposures and human colonisation with ARB. – **We agree with this important point. However, we present the results as associations and do not attempt to imply causal inference based on our findings. We have added a statement about this in our limitations section: “Additionally, several risk factors were based on caregiver-reported information and are subject to recall bias. We attempted to reduce bias by asking caregivers to recall weekly frequencies of events (e.g., child contact with animals) over longer periods of time (e.g., 3 months), rather than asking for “yes/no” responses.” (lines 784-787)**

'The primary outcomes, 3GCR-EC and ESBL-EC carriage, were assessed at the isolate level' There is no clear rationale for why the study reports AMR at the isolate level rather than the individual child (or household animal) level, especially as the methods changed part way through the study (extracting 5 E. coli isolates in the early rounds, and only 1 isolate in later rounds).- **The authors respectfully disagree with this critique. The repeated measures analysis using generalized estimating equations (GEE) and, specifically, an exchangeable working correlation structure, accounts for the imbalanced nature of our outcome measurements. If we instead aggregate our outcome measurement at the child level for each time point, the probability of assigning a child as “ESBL-EC+” would be greater for children with multiple isolates tested for the outcome. Likewise, the probability of assigning a child “ESBL-EC-“ would be greater for children with only one isolate tested for the outcome. Therefore, before beginning the analysis, we chose to include all isolates but adjust for the imbalanced number of outcome measurements per child and per time point. To test this methodological choice, we included a sensitivity analysis where we selected only the first isolate from each child to yield one outcome measurement per child per time point, which did not yield significantly different results. Other studies have also taken this approach to increase the probability of detecting the outcome, especially when the prevalence may be low (e.g., Caudell et al. 2018, The Lancet Planetary Health). We hope this clarifies our methodological choices.**

As this is intended as an epidemiological study, why is the analysis not at the individual child level, with the denominator as the total number of individuals, taking into account repeat measures? – **Building on the comment above, using GEE accounts for clustering at the individual level and allows us to describe the results (Relative Risks) and at the individual level. Using multiple isolates per time point is just another level of clustering within the individual in this epidemiological study, and does not change the interpretation of the findings.**

If the study places importance on caregiver-reported hygiene and reported infant behaviours (child handwashing, contact with animals or pets) as risk factors for child colonisation then some validity of these measures is needed. These types of indicators have poor validity & reliability in most contexts. A reporting window of child pet contact in the last 3 months (yes or no) is also a very crude indicator. – **These caregiver-reported exposures were captured in surveys using multiple choice options to improve the validity of responses. For example, when asking about child pet contact in the last 3 months, caregivers could report the number of times per week their child had contact with pets, on average, over the last 3 months. For this analysis, however, we chose to aggregate these responses into yes/no binary responses to improve power for detecting statistically significant differences in our outcomes. We recognize this is a limitation and have added a sentence on this in the discussion, as described above.**

The key limitation is the lack of assessment of antibiotic resistant E. coli in the majority of  the exposures that are reported. – **Again, we definitely agree that this is a limitation, however proxy measures are a common means of assessing exposures that are difficult to measure (due to time and financial restrains and lack of access to certain spaces – especially commercial production facilities). We did not have enough resources to assess ARB in a wide range of environmental pathways for this study, so we chose to focus on children and domestic animals. Furthermore, our proxy-level exposure measures are policy-relevant and could be used in combination with more robust data on ARB transmission pathways to support recommendations that may reduce ARB transmission (e.g., limit the number of commercial production facilities per square kilometer in residential areas).**

Other suggestions/queries.

The authors make it clear that the number of isolates per sample changed part way. However it is not clear why the analysis did not use one isolate per sample throughout all the analysis to remove the bias, then there would not be the need for a sensitivity analysis. – **We aimed to include multiple isolates per child per time point to better characterize the probability of ESBL-EC carriage for each child at each time point and capture a broader range of sequence types and genetic factors related to drug resistance. Respectfully, the goal of the sensitivity analysis was to ensure that our prespecified analysis plan did not induce bias. Because the results were similar, this validates our original analysis plan (including all isolates to capture all outcome data).**

Some of the key information for the study seems to be in supplementary tables e.g. point prevalence of child ESBL-EC colonisation at each survey, ranging from 7% to 16%, average 11%, which seems relatively low. This would merit reporting in the results and discussion. – **We have added a figure reporting the number and overall proportion of 3GCR-EC and ESBL-EC in the results – please see figure 3.**

Samples were left with participants/households for 24 hours, how was it ensured that these were actually stored in fridges or on ice? – **We did not closely monitor the overnight storage of stool samples, but based on feedback from community-based field workers we are confident the samples were stored on ice or in fridges.**

--

**Reviewer # 4**

This paper discusses the link between food and domestic animals and bacte- rial carriage in children in Ecuador.

I was asked for a statistical report and I interpret that to include all aspects of the design and conduct of the study.

# Points of detail

**Page 5** Was there a formal sample size determination to arrive at 360 house- holds? What were the stratification variables? – **We added the following sentence about stratified random sampling: “Random sampling was stratified at the neighborhood level (within parishes) based on the following strata, as determined by community-based fieldworkers: 1) backyard food animal production present in neighborhood, 2) backyard food animal production present and commercial food-animal production within 1 km of neighborhood, 3) no backyard food animal production and no commercial production within 1 km.”**

**Page 6** The finding of Amato et al here does not refer to Ecuador does it?

Perhaps clarify. **– This finding was from a study in the U.S., this has been added for clarification.**

**Page 9** There is limited information here about the categorisation of vari- ables. Categorising an essentially continuous variable wastes informa- tion (Altman and Royston, 2006; Royston et al., 2006) and leads to models which are often implausible as they predict the effect remaining flat within categories and then jumping to a new value at the category boundary. The position would of course have been worse if the choice had been made with knowledge of the data (Altman et al., 1994). – **While we respect the reviewers concerns about categorizing data, we did independently investigate whether including splines would attenuate the effect of values ‘jumping’ at the category boundary. Using splines in models did not significantly impact our results. Furthermore, using splines, especially with categorical variables with more than 2 categories, is less easily interpreted by a broad scientific audience. Therefore, we chose to maintain the categorical nature of variables, without the use of splines, in our models.**

**Page 10** Good to see the packages properly cited.

**Page 11** I am not clear about how the final figure of 1940 observations arises. It seems to exceed the number of isolates mentioned earlier in the paragraph. - **With a varying number of multiple isolates per fecal sample, we ended up with 1,940 observations across the 1,677 fecal samples. The results section on page 11 has been revised for clarification: “After removing children with missing exposure, outcome, and covariate data from household surveys, 904 3GCR-EC isolates from 1,677 child fecal samples from 594 children remained. This resulted in a total of 1,940 observations (including multiple isolates per fecal sample) in the final dataset for the primary statistical analysis.”**

**Page 12** Elsewhere in the article they are called pigs so does the use of hog here imply something? Are they distinct animals in US English as in my dialect we usually only use hog to mean someone or something which consumes excessive resources? – **Our interpretation of the distinction between hog and pig is similar to yours. We use the term ‘hog’ when referring to commercial production operations and ‘pig’ when referring to household animals (because we don’t necessarily know if the household pigs are specifically bred for production).**

**Page 12** I wonder whether means and standard deviations are best for such skewed variables. Perhaps medians and quartiles would be more repre- sentative. – **We believe standard deviations are sufficient for reporting here. We also received other comments that the results section is a bit long and dense, so we would like to keep results as brief as possible.**

**Page 14 and 15** I found some of these hard to grasp. First of all it is not always clear whether the effects, particularly the interactions, are significant. As a separate point it was only after some thought that I decided that in Table 3 strata probably means stratum. I do not have a problem with the analysis but some clarification here might be helpful. – **We have significantly revised the results section to include main effects for each food-animal exposure, distinct from interaction effects, to improve clarity. We also revised the results to be concise, removing several reports on ‘marginal significance’ to avoid confusion. We have added p values for main effects to assist with the determination for significance. For interactions, interaction p-values are presented in tables 4 and 5. Within strata in Table 3 refers to either stratum of household food animals.**

**Page 19** It is obviously undesirable, although perfectly understandable, that there was major drop–out but what exactly does the use of non

parametric measures mean and how are they supposed to have dealt with possible selection effects? – **Generalized estimating equations can generally handle missing longitudinal data assuming data are MCAR (missing completely at random), but when the probability of missing data are associated with prior values of the dependent variable, the parameter estimates may be compromised (Zorn 2001, Ballinger et al. 2016). We this early on in our analysis and found no association between the probability of our outcomes (dependent variables) and probability of missingness. We are therefore confident that GEE is an appropriate nonparametric method for dealing with possible effects of loss to follow-up, though some selection bias may remain.**

**Page 20** The phrase ‘are needed in LMICs’ implies the authors think upper– income countries are blameless. I wish I could be so confident about my own country. – **Definitely true! We 100% agree that high income countries (including our own, which fails to enforce any policies around antibiotic use or waste management in commercial food animal production) are plagued with these issues, as well. We have added “and elsewhere” after LMICs.**

**Figure 1** What does the inset show us? Is it possible to superimpose the parish boundaries as it is hard to read this one and Figure 2 together? – **This is a good suggestion, though we prefer to keep the figure as is to avoid over-complicating the map. We have added this sentence to the figure caption: “The inset is a satellite image of commercial poultry production facilities in the study area.”**

**Figure 2** This is one of the few places where pie charts might be useful. – **Thank you!**

**Figure 3** We could use a bit more explanation somewhere about what no- tations like C 500m + HH*^∗^* mean. – **This figure has been removed, as we did not feel it added any information beyond the existing results tables and was, indeed, a bit confusing.**

**Supplementary figure S1** This is an excellent idea to show us the under- lying theory but household members usage of antibiotics does not seem to be among the risk factors presented earlier (in Figure 4 for instance). – **Yes, this is true. However, these covariates were prespecified, as is standard in epidemiological analyses, so we did not remove covariates from models simply because they were not associated with the outcomes, as we expected.**

# Points of more substance

## Design

I am not clear about how the design maps on to the scientific and public health question.

- Why was it decided to use a repeated measures design when secular effects seem to be of limited importance? It would have been simpler to sample five times as many households once. Secular effects are men- tioned on pages 11 and 12but the discussion on page 17 is couched in terms of COVID–19 which can hardly have been an issue at the design stage. – **We are assessing longitudinal carriage of ESBL-EC and specific strains of E. coli in other analyses. The study was intended to be a cohort study, but due to the context within the study site (highly mobile population), we quickly realized this study would be more of a repeated measures design with additional enrollment at each time point. Covid significantly exacerbated this. To clarify, we have added the following sentence: “Prior to the pandemic, there was some dropout to do migration out of the study site.”**
- Why choose these contiguous parishes? Would there not have been more variety if they were more separated? – **There is some variety within the parishes in terms of both exposures to household and commercial food animals. However, this is a good point, because we think we would have seen stronger effects if our control (no exposure) groups were further away from this region with significant commercial food animal production.**
- It is hard to find information about the parishes but the sample sizes mentioned in Table 1 do not appear to bear any obvious relationship to the populations mentioned in the Wikipedia articles (of those which have such an article) linked from https://es.wikipedia.org/wiki/ Distrito_Metropolitano_de_Quito.- **You are correct, we did not stratify sampling based on parish and did not attempt to weight our sampling by parish population.**

Incidentally when I read the title I was confused as I did not expect a major South American city to have food production. It does become clearer in the text that the parishes are within the canton of Quito or the metropolitan region not Quito cityitself.- **This is a good point, we have revised the title to remove Quito and just include “Ecuador” to avoid confusion.**

## Context and generalisability

After reading this I do not feel I have much grasp on the way of life of these people and their animal husbandry. This would be important for people to assess generalisability to other regions of the world. Is this a region of subsistence farmers or do most householders have work outside the household and supplement their diet it with home–grown produce? Are they raising the animals for meat or for eggs or milk since given which end of the animal eggs and milk come from the risks of *E coli* must be different than from meat. – **While we recognize that context and generalizability can be further supported with descriptions of local way of life, we feel that our manuscript is already lengthy and prefer not to add descriptive text on this subject. We have done our best to provide two long tables with descriptive characteristics of households, caregivers, and children and believe this is sufficient for the current manuscript.**

## Dogs

Much of the article refers to food animals but Supplementary Table S3 shows that the predominant species for stool samples was dogs which I assume are companion animals or working dogs not a food source. What role do dogs play in the transmission? – **This matter is already discussed in lines 694-700 and lines 730-746 in the discussion section.**

# Summary

Given my background in epidemiology it is perhaps not surprising that I found this rather lacking in detail about the human beings or the animals compared with the detail on bacteria.

Michael Dewey

# References

D G Altman and P Royston. The cost of dichotomising continuous variables.

*British Medical Journal*, 332:1080, 2006.

D G Altman, B Lausen, W Sauerbrei, and M Schumacher. Dangers of using ‘optimal’ cutpoints in the evaluation of prognostic factors. *Journal of the National Cancer Institute*, 86:829–835, 1994.

P Royston, D G Altman, and W Sauerbrei. Dichotomizing continuous predic- tors in multiple regression: a bad idea. *Statistics in Medicine*, 25:127–141, 2006.
