# Supplementary material for: Risk factors for extended-spectrum beta-lactamase (ESBL)-producing E. coli carriage among children in a food animal-producing region of Ecuador: A repeated measures observational study
Source: PLoS Med. 2023 Oct 13;20(10):e1004299. doi: 10.1371/journal.pmed.1004299 (PMC10621961; doi:10.1371/journal.pmed.1004299)
Supplement: Attachment — Submitted filename: Round 3 reviewer comments.docx [file pmed.1004299.s006.docx]

**PMEDICINE-D-22-02768R3**

Editor Comments and Responses – Round 3

1. The identification of the *E. coli* isolates at the species level was not confirmed by a proper methodology.

   **The reviewer is correct that MacConkey agar followed by Chromocult agar is not enough to determine species level. However, we sequenced all these isolates and the sequences confirmed that the isolates were *E. coli*. We have included the accession numbers for all of the DNA sequences from the *E. coli* strains in the manuscript (lines 327-328).**
2. The authors used an outdated version of CLSI (2014) for interpreting the antimicrobial susceptibility results. There is an updated version freely available.

   **We incorrectly cited the year of the CLSI guidelines used in this study. This study launched in 2018 and we used the 2018 version cited below throughout our study period (2018-2022).

   *Clinical and Laboratory Standards Institute. 2018. Performance standards for antimicrobial susceptibility testing; 27th informational supplement. M100-S28. Clinical and Laboratory Standards Institute, Wayne, PA.***

   3-5. 3) The methodology employed for confirming the ESBL phenotype. 4) They use only ceftazidime disks combined with clavulanic acid. The disproportion between the number of isolates resistant to third-generation cephalosporins and the number of isolates detected as ESBL producers. In carbapenem-susceptible isolates, the production of ESBL and/or AmpC are the main mechanisms of resistance to third-generation cephalosporins in Enterobacterales. Due to the methodology employed, many ESBL-producing isolates could have been missed. It may be true because ESBL encoding genes were detected in "ESBL negative isolates" as explained below. 5) The detection of ESBL encoding genes such as the blaCTX-M genes among isolates classified as non-ESBL producers (ESBL-negative; Table S5 and Figure S3) is a cause of concern. How do the authors justify the presence of the ESBL encoding gene and the absence of the ESBL phenotype? Were the *E. coli* isolates not detected as ESBL-positives because there was an association with other resistance mechanisms? Was there a lack of gene expression? The gene may not be expressed in some isolates, but it would be uncommon to a high proportion of isolates unless there is a spread of a specific clone. Could this result be justified by the methodology employed to confirm the ESBL phenotype? The authors employed ceftazidime discs combined with clavulanic acid. It would not be a problem for screening some variants of CTX-M, such as CTX-M-3 or CTX-M-15, but it would be for other variants because cefotaxime and ceftriaxone are the preferred substrates for other variants. The authors must clarify the reasons for detecting ESBL-encoding genes in ESBL-negative isolates. Do these isolates express or not these genes? If these genes are expressed, these E. coli isolates must be reclassified as ESBL-positives. The numbers would change and new analyses would be necessary.

        **It's true that the CLSI guidelines indicate the use of CAZ - CAZ/CLA; AND CTX - CTX/CLA for ESBL testing since some isolates could show a ≥5mm difference for CAZ/CLA - CLA and not for CTX/CLA - CTX or vice-versa (i.e. a ≥5mm difference for CTX/CLA - CTX and not for CAZ/CLA - CAZ). According to CLSI guidelines, however, if a ≥ 5-mm difference in at least one combination (CAZ/CLA - CLA or CTX/CLA - CTX) is identified, it is correct to consider the isolate tested as an ESBL-producing isolate. Some ESBL-negative isolates reported in the manuscript had the potential to be ESBL-positive if we also had evaluated CTX - CTX/CLA too. Based on CLSI guidelines and methods used in this study, all of the ESBL-positive isolates detected and reported in our manuscript are ESBL-positive. The difference between the number of isolates with ESBL genes and isolates with ESBL expression (phenotypically resistant) may be, as the reviewer mentioned, due to the methodology used (only using CAZ - CAZ/CLA and not CTX - CTX/CLA). Also, there are some genetic factors that may have impacted levels of gene expression; the presence of resistance genes doesn't translate always in phenotype.**

6.	The authors observed colonization of children (Table S2 - N=5) and animals (Table S3 - N=2) by *E. coli* resistant to imipenem. The authors did not make any comments on this. Were they really *E. coli*? Was this resistance phenotype confirmed? If it was, were these isolates producers of carbapenemases? It would be interesting to know if any children’s family members had been recently hospitalized.

**We did identify 5 children and 2 animals with phenotypically imipenem-resistant isolates (confirmed *E. coli*) via AST. The following has been added to the results section (lines 437-439) to emphasize this significant finding: “**Notably, five children (<1%) from different households had one *E. coli* isolate that was phenotypically resistant to the carbapenem drug, imipenem (Table S2). All of these imipenem-resistant isolates were multidrug-resistant to four or more drug classes, with each isolate also expressing resistance to ampicillin and cephalosporin antibiotics.”

**We did not, however, identify any carbapenemases in our analysis of whole-genome sequences. Two of the five children had sought medical care from a pharmacy or private clinic in the last 3 months for an infection, but none had visited a hospital or other type of health care facility.**
